# A structured participatory preference-tuning and VR-based framework for age-friendly spatial configuration: quantifying short-term psychophysiological responses and immediate affective states through dynamic virtual environment adaptation

**DOI:** 10.3389/fpsyg.2026.1826218

**Published:** 2026-06-11

**Authors:** Jintao Zhu, Wei song Gao, Lei Wang, Yuanyuan Zou, Jiacan Wang, Ding Yuan, Na Qian, Rui Guo

**Affiliations:** 1 Department of Design, School of Fine Arts and Design, Hebei Normal University, Shijiazhuang, China; 2 Department of Design, School of Architecture and Art Design, Hebei University of Technology, Tianjin, China; 3 Tongling Polytechnic, Tongling, China

**Keywords:** age-friendly design, co-design, current emotional state, empathy map, virtual reality technology

## Abstract

The global population is aging at an increasing rate, and appropriate environmental design is crucial for the mental well-being of older adults. However, the associations between spatial configuration and psychological well-being in older adults remain insufficiently understood. To this end, a methodology integrating VR with structured participatory preference refinement was hereby proposed to assess the short-term psychophysiological responses and immediate affective states of older adults under simulated spatial configuration changes. Through conducting two semi-structured interviews with 50 elderly individuals, seven key design characteristics were identified, including ceiling height, spatial openness, natural light intensity and indoor noise levels. These insights were then translated into quantifiable experimental variables using empathy mapping and dual-cycle participatory design methodologies. Subsequently, a randomised experiment involving 18 VR scenarios was designed and 40 elderly individuals were selected as participants. Their physiological parameters (skin conductance level and heart rate) and psychological responses (measured using PANAS) were recorded. The results provide preliminary exploratory evidence suggesting associations between natural light intensity, indoor noise levels, spatial openness and older adults’ short-term psychophysiological responses. These findings should be interpreted as hypothesis-generating rather than confirmatory, due to the non-independence of repeated-measures data and the exploratory nature of the orthogonal screening design. These results align broadly with prior literature, and the primary contribution of this study lies in its integrated methodological workflow. This study identified significant associations between spatial layout and short-term psychophysiological responses as well as immediate affective states in older adults. It supports the feasibility of integrating VR and participatory design, and provides observed associations and plausible interpretations for future age-friendly environment research.

## 1 Introduction

The 21st century has witnessed an accelerating trend of global population aging. As indicated in the World Health Organization’s 2021 Report on Global Aging, the number of people aged 60 years and older will increase from 730 million to more than 2 billion between 2020 and 2050. By 2030, the elderly population in China will reach 380 million, entering a severe aging stage. Against such a backdrop, creating a living environment that allows the elderly to thrive during the aging experience while the elderly population grows, especially those threatened by poverty and unsuitable living environments, is key to China’s aging policy agenda (Li and Zhang, 2021). However, a previous study revealed that 28% of elderly individuals in China were experiencing loneliness as a prevalent psychological condition (Luo and Waite, 2014). Due to the one-child policy, most couples have only one child, which poses loneliness challenges and risks for the elderly. Retirement, solitary living, and insufficient care from offspring can exacerbate loneliness among the elderly (Hu and Wang, 2023; Jeste et al., 2020). This change in social structure triggers mental health problems and highlights the importance of an aging-appropriate physical environment as a carrier of emotional support. Improving the mental health of middle-aged and elderly people holds considerable significance for healthy aging (Lin et al., 2024).

Over the past ten years, following the implementation of the Healthy Aging strategy, the WHO has not only placed the building of age-friendly environments on the global public health priority agenda but also clarified the multi-dimensional factors affecting health and well-being, stressing that these factors are closely intertwined with individual behavior patterns and the surrounding physical environment. Against the backdrop of rapid urbanization and population aging, the mental health of the elderly has emerged as an urgent challenge for economic and social development (Shah et al., 2008). Compared with personal physical capacity, the built environment has a more profound impact on the mental health of the elderly (Zhou et al., 2022). A well-designed built environment has become the key to improving the residential satisfaction and subjective well-being of the elderly (Tang et al., 2021). The spatial layout of buildings affects the mental health and overall well-being of older people, especially those with physical disabilities (Kim et al., 2021). “Unsuitable” building spaces, such as inadequate lighting, high noise, and limited permeability, can lead to anxiety, depression, and social isolation among the elderly (Firdaus, 2017). Well-designed, light-filled, natural elements and accessible built environments promote a sense of security and well-being (Shach-Pinsly, 2019). At present, many residential areas are facing “double aging,” that is, the aging of the permanent population and the deterioration of physical infrastructure (Sun et al., 2020). This exacerbates the life difficulties of the elderly, while affecting their mental health and social participation.

Current research has demonstrated that age-friendly environments are positively associated with both physical and mental health in older adults, with a more pronounced impact on their psychological well-being. The number of environmental factors moderates their association with physical health (Zhou et al., 2022). Within long-term care facilities, the majority of elderly residents express satisfaction with the homelike environment and privacy provisions. However, some are dissatisfied with privacy conditions, whilst sleep quality is affected by sensory environmental factors and visitor activity. There is a widespread desire for more spacious rooms, necessitating optimised environmental design to enhance their quality of life (Bae and Kim, 2024).

From the perspective of environmental psychology, older adults’ psychophysiological responses to spatial environments are guided by mature theoretical foundations. First, environmental stress theory suggests that physical environmental stimuli (light, noise, spatial openness, crowding) exceed an individual’s adaptive capacity, triggering sympathetic nervous system arousal and negative affect. Second, prospect-refuge theory explains that spatial openness and visual permeability shape safety perceptions: semi-open structures provide both visibility and shelter, reducing anxiety. Third, sensory and cognitive aging theory highlights that older adults have reduced tolerance for noise, dim light, and crowded spaces, making them more sensitive to environmental stimulation. Fourth, participatory environmental design theory emphasizes that real user needs must be embedded into variable design to ensure ecological validity. Despite these theories, existing research rarely systematically integrates them into a unified framework to guide variable selection, experimental design, and multimodal measurement. Most studies remain descriptive or cross-sectional, lacking a theoretical path from spatial features, psychological processes, physiological responses, to affective states. The present study is therefore theoretically positioned to fill this gap by constructing a theory-driven, VR-based, structured participatory feedback and preference-tuning approach that explains how and why spatial configuration influences older adults’ immediate responses.

Based on the above theory, this study established a clear and verifiable conceptual model to serve as the core organizational logic of the entire study: spatial environmental characteristics—perceived environmental stress/safety—physiological arousal (SCL/HR)—immediate emotional state (PA/NA). This model was established before the experiment began. All variables, virtual reality procedures, hypotheses, and analyses were directly derived from this model.

In recent years, the application of immersive technologies in the fields of elderly health and environmental design has garnered increasing attention, and relevant research has established a clear consensus on age-friendly design. Through iterative user-centered design and think-aloud experiments, Lundstedt et al. (2023) proposed that virtual natural environments for older adults should adhere to three key principles: authenticity, interactivity, and relevance. They emphasized that virtual environments must possess high visual realism, low cognitive load, and the ability to evoke nostalgic memories, and demonstrated that seated navigation and simplified interactions can significantly enhance the safety and comfort of VR use among older adults. Meanwhile, Ullal et al. (2024) developed a collaborative augmented reality activity through six rounds of iterative participatory design, further confirming that older adults prefer simple, nostalgic, and low-cognitive-load interaction forms (Lundstedt et al., 2023). Standard gesture-based interactions present significant barriers for older adults with declining physical function. Meanwhile, voice assistance and simplified interfaces can greatly enhance usability. Additionally, the realism of avatars and interface legibility directly affect user acceptance. Together, these two studies demonstrate that the age-friendly adaptation of immersive technologies must focus on users’ genuine needs. It should transform qualitative preferences into quantifiable, actionable design parameters through a participatory process.

While existing research has demonstrated that the layout of the built environment significantly impacts the mental health and overall well-being of older adults, current studies are still subject to three core limitations: First, previous studies have predominantly employed a univariate analytical framework, lacking systematic screening and quantitative comparison of the independent main effects of multidimensional spatial factors. Furthermore, due to the absence of objective physiological data, it has been difficult to comprehensively identify the core spatial factors influencing the mental health of older adults. Research has typically overlooked the moderating role of older adults’ socioeconomic status and cultural background in spatial perception (Yang and Fu, 2019). This one-dimensional research paradigm struggles to reveal the combined associations of multiple interacting factors with mental well-being of older adults, resulting in an incomplete understanding of how spatial layout exerts its influence. Secondly, there is a lack of objective physiological data. Most existing studies use cross-sectional designs, making it challenging to tell the difference between the long-term cumulative effects of spatial layout and the short-term effects of stimuli. When investigating the impact of spatial layout on the mental well-being of older adults, a lack of objective physiological data also impedes the validation of the potential links through which residential environments may relate to psychological and physical outcomes at an individual level. Thirdly, elderly user involvement remains insufficient. Specifically, most studies treat older adults as passive information providers rather than active participants in the design process. Existing engagement models predominantly rely on one-off cross-sectional surveys, failing to establish a collaborative framework that spans the entire design lifecycle. This restricts the ability to address the specific needs of community-level research into the mental well-being of older adults (Ji et al., 2023). Most previous studies either lack a clear conceptual model or use theory solely for ex post facto interpretation. Few studies employ a theoretical model as the organizing logic that runs throughout the entire study.

Beyond these methodological limitations, a fundamental theoretical gap exists: existing research has not established a clear conceptual model that links spatial characteristics to psychological processes in older adults. It remains unclear why certain spatial features matter, through which cognitive or sensory mechanisms they operate, and how participatory insights can be translated into testable theoretical propositions. This study addresses this theoretical gap by building a conceptual framework from the outset, rather than applying theory only after results are obtained. In addition, existing VR-based environmental studies often lack direct assessments of environmental realism, perceived presence, and ecological validity, making it difficult to confirm whether experimental responses can be transferred to physical age-friendly environments.

In order to address the aforementioned research gaps, particularly the lack of methods integrating user preference elicitation with controlled experimental validation of multi-factor effects, this study proposed and verified an innovative methodological pipeline. This pipeline integrates participatory requirements gathering (to identify and prioritize key design variables) with VR-based controlled experimentation (to efficiently test the main effects of these variables), while concurrently collecting multi-modal data (objective physiological SCL/HR and subjective psychological PANAS) for a more holistic assessment. This research methodology aims to provide a more systematic and ecologically valid approach to investigating the effects of spatial configuration on short-term psychophysiological responses and immediate emotional states in older adults, thereby complementing traditional survey or post-occupancy evaluation methods. However, this study focuses on immediate responses to brief spatial exposure in a VR environment, using physiological indicators (SCL/HR) and emotional scales (PANAS) for measurement. These indicators reflect only short-term psychophysiological changes and immediate emotional states; they are not equivalent to mental health in clinical or long-term contexts, and conclusions cannot be directly extrapolated to the effects on mental health associated with long-term residency.

## 2 Materials and methods

### 2.1 Participatory design

Prior to commencing the participatory design process, an interview was initially carried out with the elderly population to analyze and screen potential participants. This research adopted a two-stage stratified sampling strategy to strike a balance between ecological validity and experimental control. Through stratified sampling, 50 participants (including 24 females) were selected from a community in Shijiazhuang. The target group was composed of long-term independently living older adults. They had authentic and abundant experiences in spatial utilization and daily life, which made them appropriate for identifying latent needs and extracting core spatial design variables. Their spatial habits, preferences, and sensory requirements could mirror the general demands for age-friendly spaces among the elderly.

The participants in the qualitative interviews of this study were independently living older adults in the community, while those in the VR experiments were older adults residing in long-term care facilities. These two groups differed not only in recruitment contexts but also in conceptual aspects such as daily-life autonomy, environmental control ability, daily routines, spatial demands, and environmental dependence degree. Community-dwelling older adults had a higher degree of environmental autonomy, whereas those in long-term care facilities were more reliant on the safety, stability, and support provided by their environment. Consequently, there might be systematic disparities in their spatial perception and preferences.

**Figure 1:**
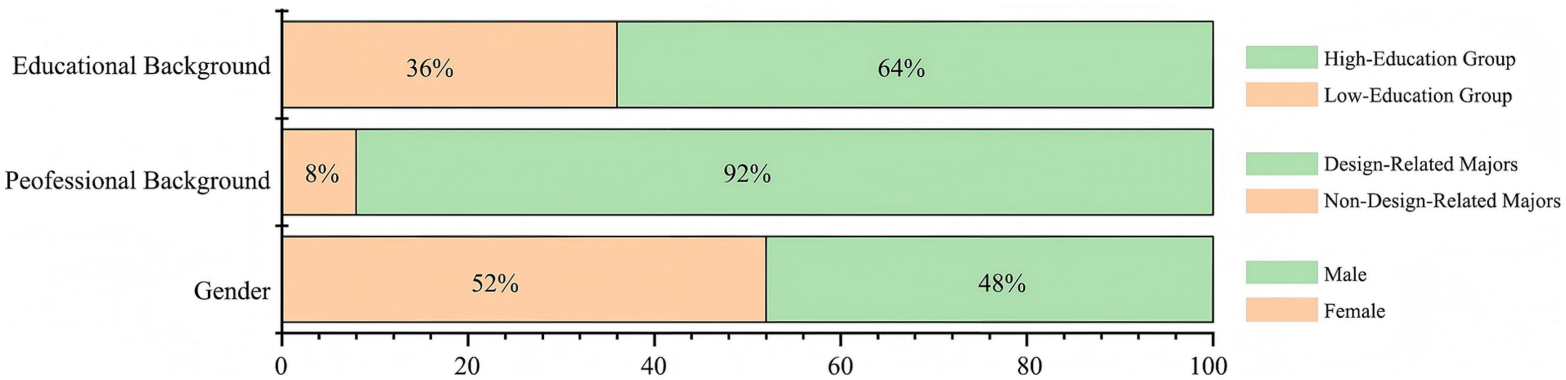
Demographic characteristics of respondents.

**Figure 2:**
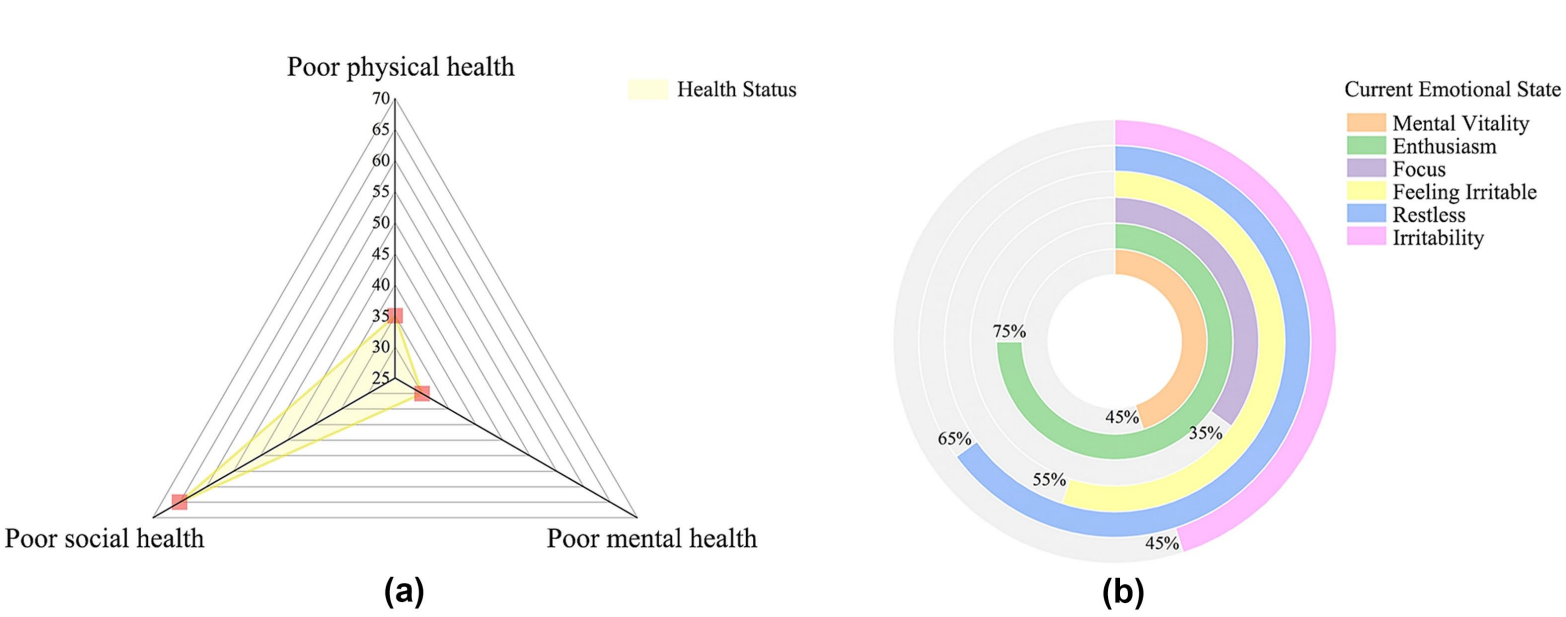
Healthy and mental condition statistics: **(a)** Statistics on respondents’ health condition; **(b)** statistics on respondents’ mental condition.

To guarantee demographic equilibrium across gender, professional background, and educational attainment, the 50 participants were partitioned into distinct stratified groups in accordance with the following criteria: Professional background was classified based on work or academic experience. Individuals from the domains of architecture, urban planning, and environmental design were allocated to the design-related group, whereas all others formed the non-design-related group. The educational background of the population was defined with reference to the development of higher education in China over the past century. Qualifications at or above the secondary school level (including technical secondary schools) demarcated the higher education group, while those below this level constituted the lower education group. Figure 1 depicts the demographic characteristics of the respondents. Prior to the initiation of the first interview, the PANAS scale was utilized to determine the physical and mental states of the participants, with statistics on their physical and mental conditions presented in Figure 2.

**Figure 3:**
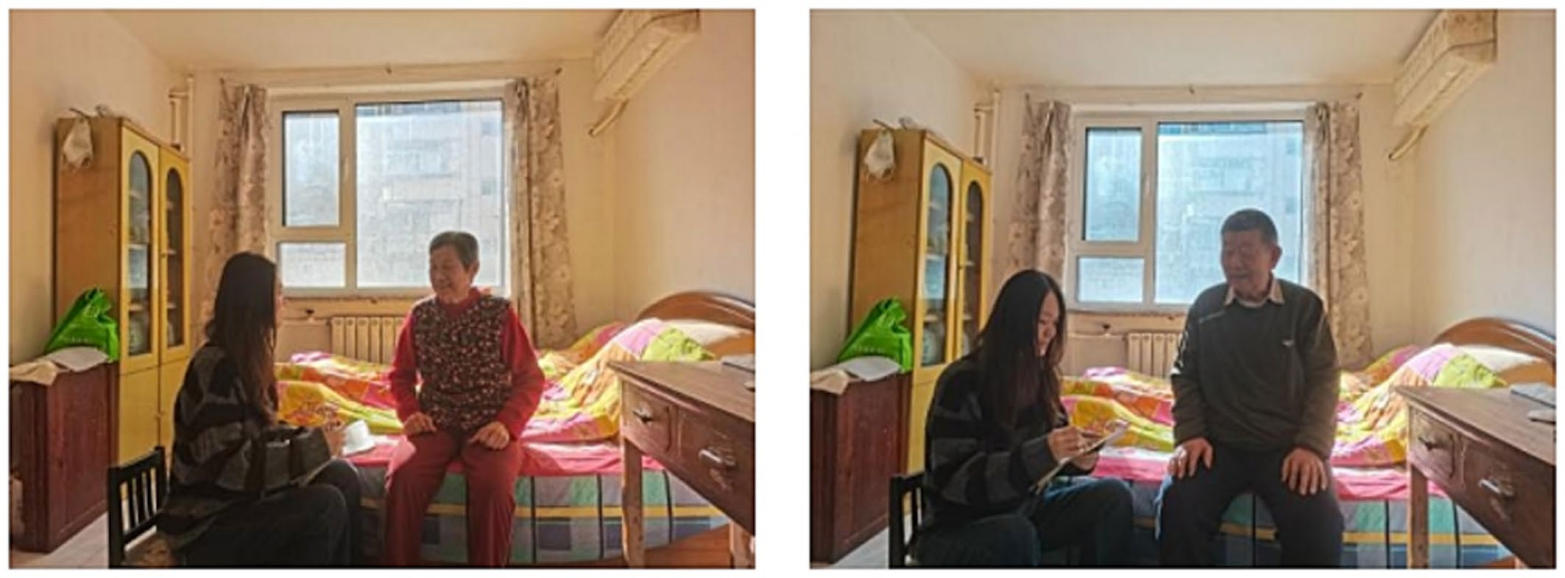
Scene of researchers interviewing elderly individuals.

Semi-structured interviews were conducted in tranquil and comfortable environments to enable participants to fully articulate their viewpoints. The scenario of researchers interviewing the elderly is presented in Figure 3. Before commencing the formal interviews, the research team offered detailed elucidations regarding the research aims and content, and only proceeded after obtaining informed consent. To amass more comprehensive Supplementary Data, follow-up interviews were conducted with a subgroup of participants.

**Figure 4:**
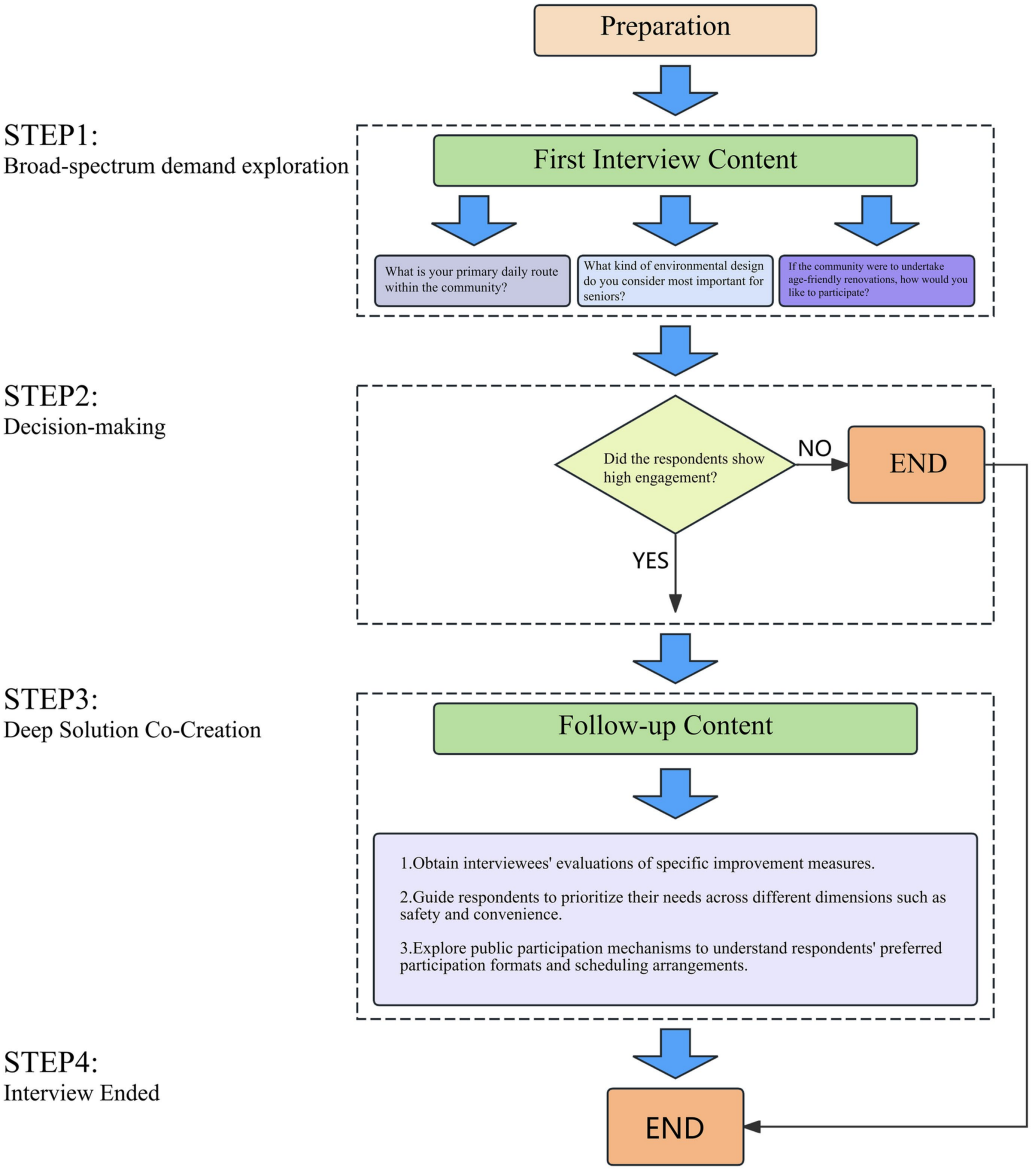
Interview procedure and content.

The initial interview with the respondents encompassed general inquiries, systematically amassing viewpoints concerning three aspects: the experiences of senior citizens with age-friendly facilities, design preferences, and the willingness to participate. This furnished empirical evidence for the subsequent experiments. The second-round interviews were directed at design-related majors who exhibited a high level of engagement during the initial interviews. Adopting a thematic, semi-structured approach, these sessions were based on the core topics of the first round, while concentrating on exploring design improvement solutions and participation mechanisms with higher practical value. The interview procedure and content are presented in Figure 4.

**Figure 5:**
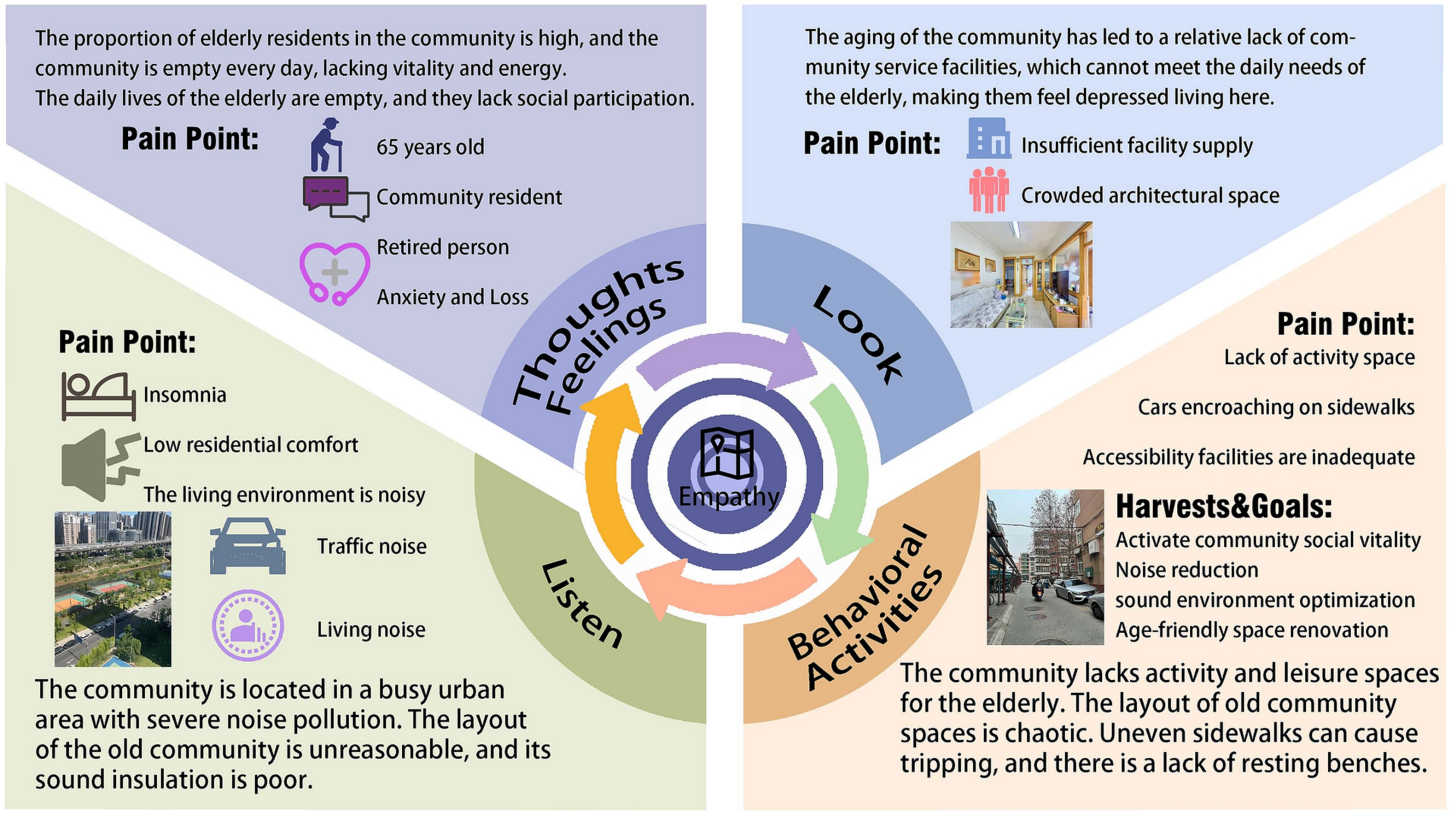
Empathy map construction.

**Figure 6:**
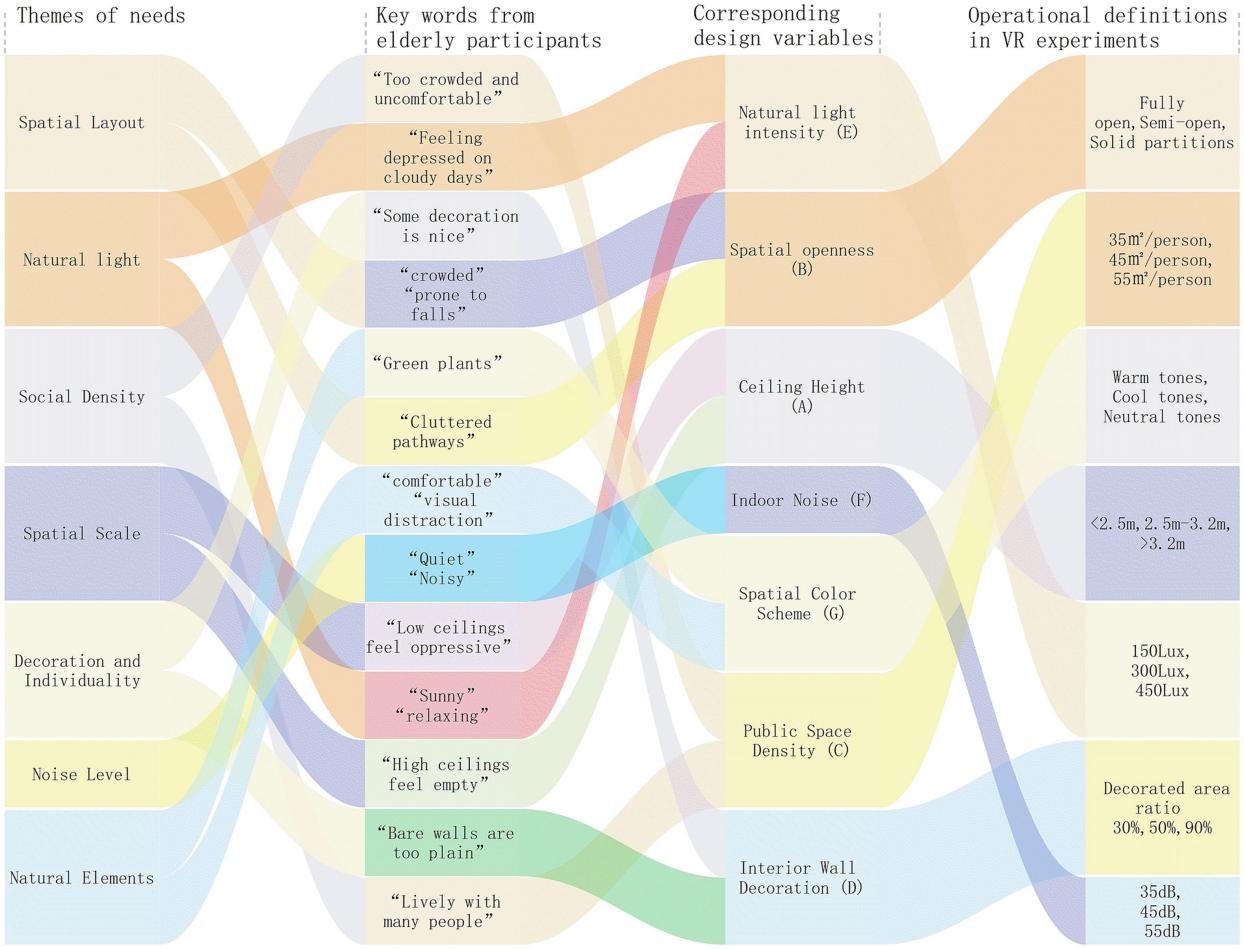
The transformation of latent requirements.

In the participatory design for the elderly, an empathy-building approach was adopted, during which an empathy map was employed to discern their unarticulated demands. Figure 5 presents the empathy map, which was developed on the basis of interviews with the elderly and incorporated elements such as sensory experiences, visual perceptions, and auditory cues. Through the analysis of two interview sessions, both the explicit and implicit needs of the elderly were translated into seven quantifiable design variables. These seven identified variables were not only derived from empirical interviews but also in line with the four theoretical foundations. Figure 6 depicts the transformation of latent requirements.

This study utilized thematic analysis to systematically code data from two rounds of interviews. Firstly, two researchers independently carried out open-coding of the interview transcripts to extract raw concepts associated with spatial perception, sensory experiences, and comfort needs. Subsequently, these concepts were categorized via axial coding to form higher-order need themes. Finally, through selective coding and theoretical saturation testing, the core need dimensions were identified. To guarantee the reliability of the analysis, this study adopted a double-blind coding approach by two researchers, with the coding consistency exceeding 0.85, indicating a high degree of reliability. The coding results were reviewed and validated by a third expert in the field of environmental design, thus eliminating subjective bias.

Herein, a “dual-cycle participatory design model” was adopted, with professional designers leading the structural design, while elderly users participating in optimizing details (Liddicoat and Newton, 2019).

**Figure 7:**
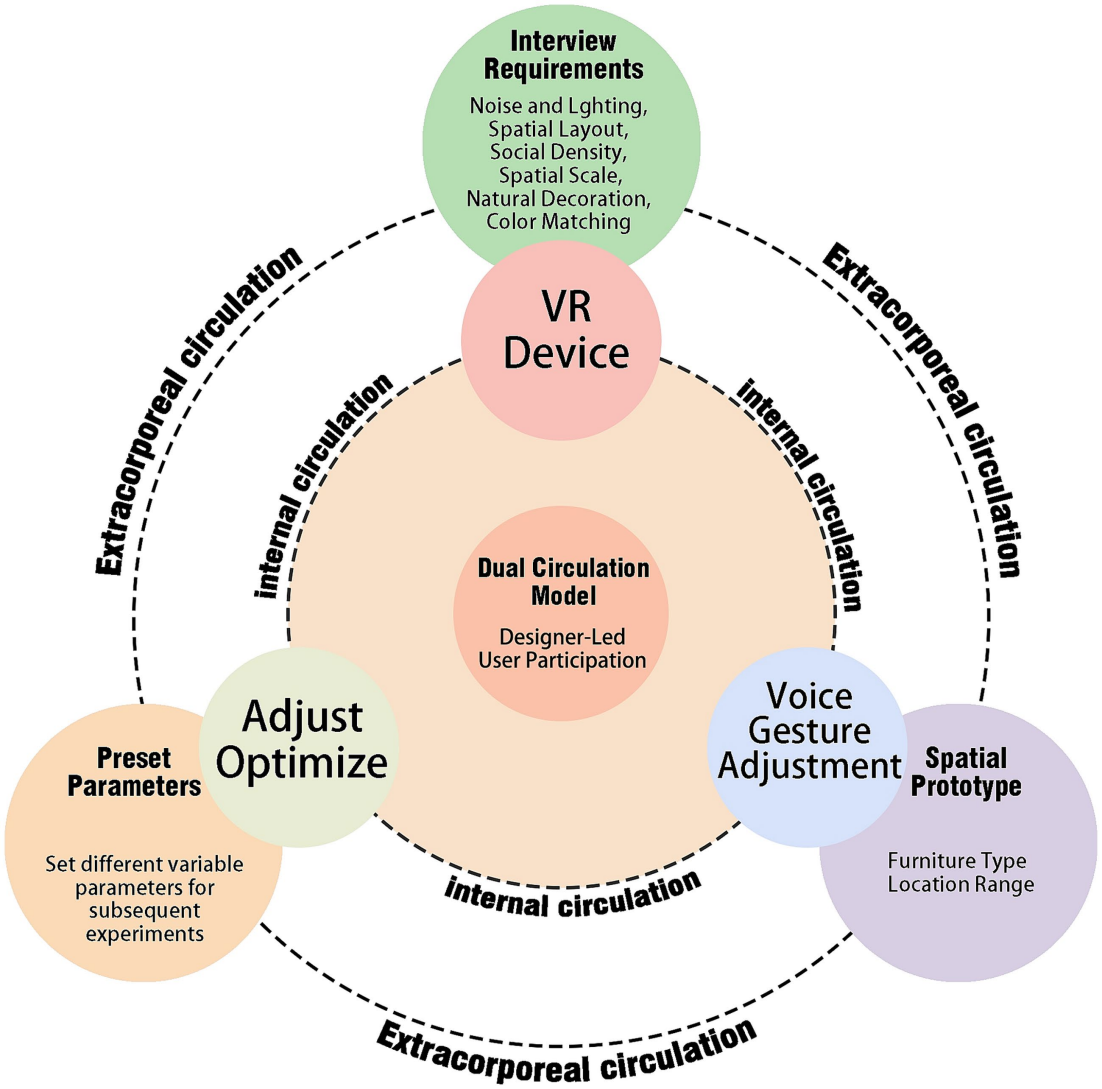
Dual-cycle participatory design model.

The dual-cycle participatory design model is presented in Figure 7. The external loop was spearheaded by the research team, which translated interview requirements into an initial VR spatial prototype and established adjustable design parameter ranges. The internal loop invited elderly users to conduct real-time optimization settings within these parameters through VR devices. The VR environment functioned as an effective “common language” for communication between professional design knowledge and user knowledge. Elderly participants accessed the initial virtual space via VR headsets. Participants were permitted to adjust seven predefined environmental parameters in real time using natural voice commands and simple gestures. The system concurrently recorded their operation frequency, duration of lingering, and voice keywords to capture their implicit preferences. Relevant data are detailed in Appendix A. Ultimately, each participant determined their most preferred “digital prototype” spatial configuration, and this personalized data guided the selection of variable levels in subsequent orthogonal experiments.

The conceptual framework, experimental paradigm, and major categories of variables in this study were all predefined by the research team based on theoretical considerations, constituting a participatory preference calibration within a closed-ended structure. Elderly participants did not contribute to the formulation of research questions, the construction of the theoretical framework, or the design of the experimental paradigm. Their core contributions involved identifying high-frequency pain points from a predefined list of spatial elements, prioritizing needs, and determining variable levels and acceptable thresholds. Therefore, the participatory design of this study should be characterized as structured preference optimization rather than open, egalitarian co-design.

### 2.2 Participants

To attain the research objectives, a purposive sampling approach was adopted to select a total of 40 elderly individuals (20 males and 20 females) aged 60 and above from a nursing home. This method is mainly utilized for standardized control experiments and the collection of physiological signals. In nursing homes, the elderly have regular daily schedules, a consistent living milieu, and fewer external interferences, which effectively improves the stability of physiological indicators such as skin conductance level (SCL) and heart rate (HR), thus reducing experimental bias. The target population comprised elderly volunteers who could express their subjective experiences fluently and were in good physical condition, without being diagnosed with visual impairment, attention-deficit/hyperactivity disorder (ADHD), autism spectrum disorder (ASD), or claustrophobia. The elderly samples were grouped according to the Mini-Mental State Examination (MMSE) cognitive assessment scores to differentiate their degrees of cognitive decline. All participants scored ≥ 21 on the MMSE and could articulate their subjective experiences fluently. They underwent MMSE assessment and were stratified into two groups based on scores (Normal: ≥26 points; Mild Impairment: 21–25 points). After MMSE stratification (Normal/Mild Impairment), participants were randomly assigned within each stratum to form 8 groups. This study was carried out in accordance with the Declaration of Helsinki and was approved by the Ethics Committee of Hebei Normal University. Each participant read and signed an informed consent form before the experiment.

### 2.3 Experimental design and variables

Through systematic thematic analysis, dual-coding validation, consensus-based decision-making rules, and a two-phase participatory design process, this research identified seven crucial physical environmental characteristics. These characteristics directly originate from the consensus requirements of older adults rather than the assumptions of researchers. These variables were not merely selected via theoretical screening; instead, they were determined according to the actual sensory preferences, comfort requirements, and pain points repeatedly mentioned by 50 participants. Consequently, the participatory design phase laid the groundwork for variable definition, hierarchy selection, and the establishment of the experimental framework. This study identified seven crucial physical environmental characteristics that significantly influence the short-term psychophysiological responses of older adults. Table 1 lists these variables converted into quantifiable design variables for this experiment. Multiple dimensions, including spatial scale, layout, and sensory experience, were covered to ensure the comprehensiveness and user relevance of the research elements. Building upon the specific descriptions provided by the elderly participants during interviews and feedback from the dual-cycle participatory design model, three levels of variables were established for the seven design features. Secondly, quantitative definitions were established for each design feature. Regarding spatial openness, “Fully Open” is defined as having no visual barriers; “Semi-Open” is defined as partial obstruction using wooden grids; “Solid Partitions” are defined as opaque walls matching the interior ceiling height. Quantitative definitions, visual examples and key dimensions for the three levels of spatial openness (B) may be found in Appendix B. The seven spatial variables in this study were derived from the qualitative needs of community-dwelling older adults, whereas the VR experiments were conducted with older adults in long-term care facilities. They were systematically selected based on a conceptual model to represent environmental stress, perceived safety, spatial awareness, and sensory load. Empirical testing of the consistency of spatial preferences between these two groups was not conducted. The transferability of such preferences remains a plausible theoretical supposition that lacks empirical substantiation. Consequently, whether the variables derived from the needs of community-dwelling older adults can be fully applied to older adults in long-term care facilities necessitates direct verification in future research.

**Table 1:** Design features at different levels.

Design features	Scene 1	Scene 2	Scene 3
Ceiling height (A)	Below 2.5 meters	2.5 meters to 3.2 meters	Over 3.2 meters
Spatial openness (B)	Semi-open	Fully open	Solid partitions
Public space density (C)	35㎡/person	45㎡/person	55㎡/person
Interior Wall Decorations (D)	30%	50%	90%
Natural Light Intensity (E)	150Lux	300Lux	450Lux
Indoor noise (F)	35 dB	45 dB	55 dB
Spatial color scheme (G)	Warm-toned space	Cool-toned space	Neutral-toned space

This study incorporated 7 spatial design variables at 3 levels each, resulting in a total of 3^7^ = 2,187 experimental scenarios. This far exceeds the experimental load that elderly participants can tolerate, making it highly likely to induce interference from factors such as fatigue and adaptation effects, which could render the data invalid. Orthogonal experimental designs are based on the core principles of orthogonality, balance, and representativeness. They enable precise, unbiased estimation of the main effects of each variable while significantly reducing the number of experimental scenarios. At the same time, they ensure that variable combinations cover the full range of spatial characteristics, balancing experimental feasibility with data validity. This makes them the optimal method for efficiently screening core influencing factors in multi-factor, multi-level experiments. Orthogonal experiments were conducted within the established framework of variables and levels to ensure scientific and efficient data collection (Zou et al., 2017). Although the interaction effects of spatial factors hold theoretical significance in the design of age-friendly environments, the primary objective of the first phase of this study is to efficiently identify key main effects rather than to comprehensively analyze interaction mechanisms. This study employs a within-subjects repeated-measures design, wherein observations from the same participant are correlated and do not meet the independence assumption required for standard ANOVA. Recognizing this limitation, we abstain from considering the ANOVA results as confirmatory statistical inferences. Instead, the analyses are utilized for exploratory main-effect screening, aiming to identify candidate spatial features that are worthy of further exploration via more rigorous repeated-measures or mixed-effects models in future research. All reported *p*-values and effect sizes are descriptive of the observed trends and should not be interpreted as conclusive evidence of population effects. Owing to the limitations associated with the physiological tolerance of elderly participants, the cognitive load of VR experimental scenarios, and the duration of the experiments, the conditions for performing a full factorial analysis of interaction effects are not yet established.

**Figure 8:**
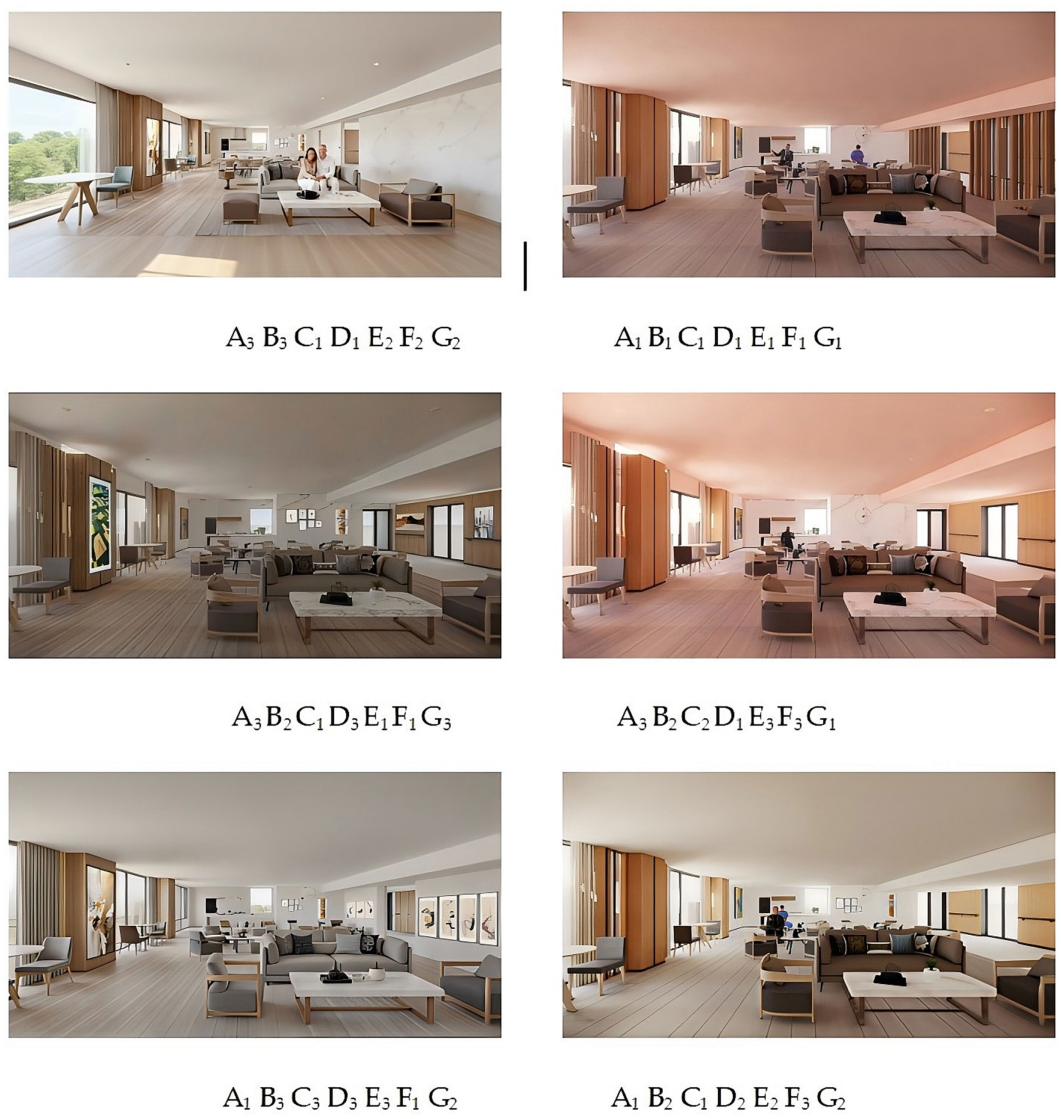
Six sample screenshots of the experimental scenario.

This orthogonal experiment is exclusively designed to examine the primary effects of the seven spatial design variables. It does not entail formal modeling or statistical testing of the higher-order interaction effects among the variables. The analysis scope is confined to identifying the core spatial factors that exert an independent and significant influence on the short-term psychophysiological responses of older adults, rather than clarifying the intricate mechanisms of multifactorial interactions. This research overcomes the limitations of previous single-factor paradigms by effectively identifying core spatial factors that have an independent and significant impact on the psychophysiological responses of older adults. This study surmounts the limitations of previous research, including the dearth of objective physiological data and inadequate user involvement, and offers quantifiable core parameters to direct age-friendly spatial design. The participatory design stage served as the “input,” while orthogonal experiments were instrumental in validating the statistically significant influence of the design elements obtained from participatory design on the indicators of the elderly. The feature combinations of these 18 scenarios are presented in Table 2, and Figure 8 depicts the visualizations of six representative scenes. Despite the two samples being sourced from distinct living environments, the seven design features identified among community-dwelling older adults are universal spatial sensory elements shared by both community-based and institutional care settings. Research on elderly environments has verified that the fundamental perceptual preferences of the elderly regarding light, sound, and spatial scale do not vary significantly across different living environments, ensuring logical coherence from the identification of needs to experimental verification.

**Table 2:** Orthogonal experimental design.

Design features	1	2	3	4	5	6	7	8	9	10	11	12	13	14	15	16	17	18
Ceiling height (A)	A3	A1	A3	A2	A3	A1	A2	A3	A1	A1	A3	A2	A1	A3	A2	A1	A2	A2
Spatial openness (B)	B3	B1	B2	B1	B1	B3	B3	B2	B1	B2	B3	B1	B3	B1	B2	B2	B2	B3
Public space density (C)	C1	C1	C1	C1	C2	C2	C1	C2	C2	C3	C3	C3	C3	C3	C3	C1	C2	C2
Interior wall decorations (D)	D1	D1	D3	D2	D2	D2	D3	D1	D3	D1	D2	D1	D3	D3	D2	D2	D3	D1
Natural light intensity (E)	E2	E1	E1	E3	E3	E1	E3	E3	E2	E3	E1	E1	E3	E2	E2	E2	E1	E2
Indoor noise (F)	F2	F1	F1	F2	F1	F2	F3	F3	F3	F2	F3	F3	F1	F2	F1	F3	F2	F1
Spatial color scheme (G)	G2	G1	G3	G3	G2	G1	G1	G1	G3	G3	G3	G2	G2	G1	G1	G2	G2	G3

Prior to conducting the experiment, the following research hypotheses were formulated on the basis of interview data and co-design findings, aiming to offer a clear orientation for the subsequent experimental procedures.

*H1:* Derived from environmental stress theory and perceived control theory, low indoor ceiling height (A1) was significantly associated with higher skin conductance levels (SCL) and negative effect (NA) in older adults.

*H2:* Derived from prospect-refuge theory, semi-open spaces (B1) showed a significant relationship with more stable heart rate (HR) and higher positive affect (PA) compared to fully open and solid partitioned spaces.

*H3:* Derived from environmental stress theory and sensory aging theory, higher natural light intensity (450Lux) and lower noise levels (35dB) was significantly associated with Lower skin conductance levels (SCL) and negative effect (NA) in older adults.

*H4:* Derived from environmental psychology of color and affect, warm-colored spaces (G1) was significantly associated with higher positive affect scores and lower negative effect compared to cool-colored spaces.

### 2.4 Experimental procedure and measurement tools

This research adopted a within-subjects repeated-measures design. Given that multiple observations from the same subject are interrelated, the assumption of independent and identically distributed data is not fulfilled. This research is an orthogonal main effects screening study with the objective of identifying key influencing factors. The analysis of variance (ANOVA) results are utilized to evaluate the trends in factor significance, and the corresponding conclusions represent preliminary exploratory findings.

**Figure 9:**
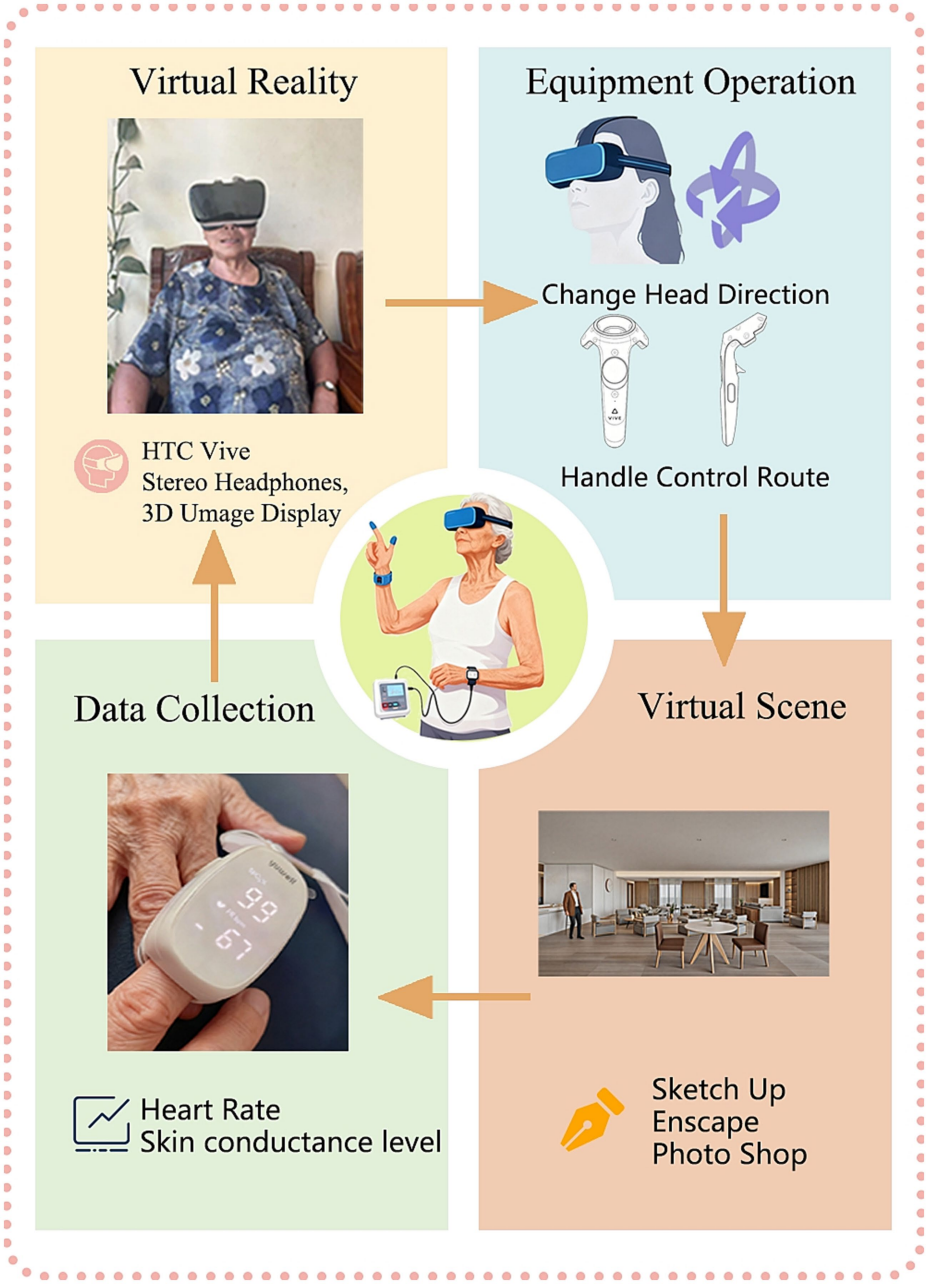
Experimental process scene.

The virtual reality (VR) experiment was carried out in a specially designed chamber, which provided a stable physical setting to present participants with virtual environments featuring various combinations of session characteristics. This research adopted a within-subjects repeated-measures design, where each elderly participant successively underwent 18 VR scenarios. A significant correlation was observed among the multiple observations of the same participant, and the data failed to satisfy the assumption of independent and identically distributed observations. The experiment was conducted from September to November 2025, with sessions carried out between 2:00 p.m. and 4:00 p.m. to mitigate the influence of lighting on physiological indicators and emotional states. Elderly participants utilized head-mounted devices to engage in immersive scene experiences. The integrated headset employed was an HTC Vive, which consisted of stereo headphones, 3D image displays, and positioning equipment. Scene models were constructed as 1:1 virtual environments using SketchUp, rendered through Enscape, and ultimately color-adjusted in Photoshop. Interaction and display were realized via 3D panoramic video within the HTC Vive. Uniform viewpoints were adopted across all experimental scene models, and participants were positioned at the same location within the virtual space to enable a comprehensive understanding of the spatial environment. Figure 9 depicts the experimental process scene. Physiological signals were recorded by means of a multi-channel physiological recorder. Skin conductance (SCL) was measured using Ag/AgCl wet electrodes placed on the index and middle fingers of the non-dominant hand of the subject; heart rate (HR) was concurrently recorded through photoplethysmography (PPG). The device had a consistent sampling rate of 1,000 Hz and a bandwidth of 0.01–10 Hz, and was equipped with built-in filtering and noise reduction modules.

**Figure 10:**
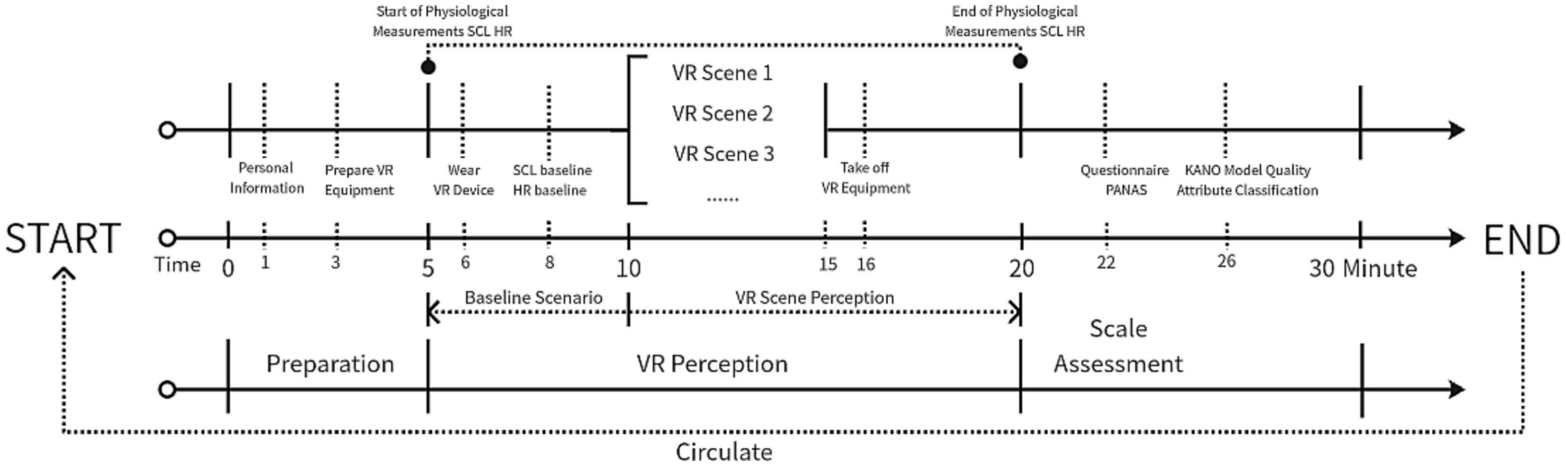
Schematic diagram of the experimental process.

The experimental protocol was composed of three phases: preliminary preparation, VR scene perception, and scale evaluation. Each experiment endured for approximately 25 min. Every VR scene persisted for 90 s to guarantee that participants could comprehensively perceive the spatial attributes without succumbing to fatigue. A compulsory two-minute rest interval was implemented between scene transitions to enable participants to revert to a stable state. No participants withdrew during the experiment, and none reported any discomfort subsequently, thereby ensuring the ethical soundness and validity of the study. The diagram of the experimental protocol is presented in Figure 10.

During the preparation phase, a volunteer guided participants into the experimental space. Participants were briefed on the experimental procedure, completed the required personal information forms, and adjusted the VR experimental equipment until the scene images were clearly visible. The comfort level of the VR equipment was confirmed with participants to ensure an immersive experience.

The VR experience phase consisted of two segments: baseline scenario and VR scenario experience.

Prior to the experiment, VR equipment and physiological monitoring devices were fitted to the elderly participants to measure their physiological indicators in both relaxed and experimental states. Throughout the experiment, participants’ skin conductance levels and heart rate data were monitored and collected in real time.

Upon the commencement of the formal experiment, participants were directed to be seated in a physically stable environment. The virtual reality (VR) display presented the pre-designed spatial environment through an orthogonal experimental design. Participants engaged in immersive observation of the spatial environment, and their physiological response data were recorded. Subsequently, the system reverted to the baseline scene, where participants were instructed to take a rest to prepare for the subsequent procedures.

Upon the conclusion of the VR experiment, participants were instructed to remove their VR headsets and physiological monitoring devices. Subsequently, they were administered the Positive and Negative Affect Schedule (PANAS) questionnaire. After verifying the completeness of the data, the equipment was cleaned and disinfected for the subsequent participant.

We employ multiple collection methods to gather outcome data, including physiological responses, psychological responses, and spatial quality assessment data. Each dataset is processed according to its corresponding methodology and guidelines.

Skin Conductance Level (SCL) serves as an indicator of sympathetic nervous system activity, operating on the principle that changes in skin electrical properties result from sweat gland activity (Li et al., 2022). A decrease in its value signifies heightened relaxation and reduced stress levels. HR denotes the number of cardiac contractions per minute (Abd-Alhamid et al., 2020). A decrease in heart rate is often accompanied by an increased sense of relaxation, whereas an increase in heart rate may be associated with tension induced by environmental factors. Therefore, These indicators are frequently employed in environmental psychology research to measure immediate psychological response and stress responses (Gao and Zhang, 2020). physiological data is determined by measuring skin conductance levels (SCL) and heart rate (HR).

Prior to conducting an analysis of the physiological effects of spatial design characteristics on the elderly, physiological indicators need to be proportionally scaled down for statistical analysis. The mean difference in Skin Conductance Level (SCL) scores before and after the Virtual Reality (VR) experience can be regarded as evidence of physiological (Gao and Zhang, 2020) alterations. The ideal data model for SCL is presented in Appendix C. The mean SCL value recorded five seconds before the participants’ baseline physiological testing will be used as the initial indicator (SCLta). The mean SCL value recorded during the last five seconds of the VR experience serves as the final metric (SCLtb). Considering that SCL is significantly affected by the baseline state of sweat glands and the sympathetic nervous system, along with notable individual variations, its response cannot be directly calculated. The calculated rate of change in SCL during the VR experience phase is shown in Equation 1. The same formula is also employed to calculate the rate of change in Heart Rate (HR) during the VR experience phase.

$$\Delta \mathrm{SCL}=(\bar{\text{SCLtb}})-(\bar{\text{SCLta}})/\bar{\text{SCLta}}$$ (1)

The PANAS scale is employed to measure changes in participants’ psychological states and emotional responses. The PANAS is a widely used and well-established instrument. Self-assessment report on the positive and negative effects of validation, originally compiled by Watson (Watson et al., 1988). Considering cultural differences, certain terms in the original PANAS scale could not be easily comprehended within the Chinese linguistic context, prompting the use of a revised version (Qiu et al., 2008). The original PANAS scale is presented in Appendix D. It is better suited to the Chinese linguistic context and the cognitive abilities of older adults. This version has been used in numerous studies on mental health among older adults in China and has demonstrated good reliability and validity. The scale includes positive affect (PA, 9 items) and negative affect (NA, 9 items), and is scored on a 1–5 point scale. In each scenario, the Positive Affect Score (PA) and Negative Affect Score (NA) are calculated as the sum of each category, serving as a measure of the participant’s psychological response. This study did not include standardized scales or quantitative assessments to measure environmental realism, sense of presence, or perceived ecological validity during VR exposure.

## 3 Results

### 3.1 Analysis of main effects for design feature

This study employed a within-subjects repeated-measures design, in which each elderly participant sequentially experienced 18 VR scenes. The following analyses were conducted to directly test the conceptual model proposed in the introduction. All results correspond to the theoretical pathways. There was a significant correlation among the multiple observations for the same participant, and the data did not meet the assumption of independent and identically distributed observations. The analysis revealed that four design characteristics showed significant statistical associations with physiological and psychological indicators of elderly individuals, namely ceiling height (A), spatial openness (B), natural light intensity (E), and indoor noise levels (F). A summary of design features with significant effect is presented in Table 3. The full version of design characteristics with significant effect is provided in Appendix E. Notably, natural light exposure (E) and indoor noise (F) not only demonstrated extreme significance across all indicators (*p* < 0.001), but also exhibited the largest effect sizes (e.g., η^2^ values of 0.367 and 0.322, respectively, for SCL). This observation identified these elements as core environmental factors influencing physiological stress responses in older adults. Hypotheses H1, H2 and H3 were thus confirmed, whereas H4 (concerning colour schemes) was not supported.

**Table 3:** A summary of design characteristics with significant effect.

Variable	Design characteristics	Df	Mean square	F	*p*-value	η^2^
SCL	Natural light intensity (E)	2	1.746	33.901	<0.001*	0.367
	Indoor noise (F)	2	1.583	27.781	<0.001*	0.322
HR	Spatial openness (B)	2	107.200	26.321	<0.001*	0.310
	Natural light intensity (E)	2	104.358	28.746	<0.001*	0.330
	Indoor noise (F)	2	98.775	19.934	<0.001*	0.254
PA	Ceiling height (A)	2	74.308	9.671	<0.001*	0.142
	Spatial openness (B)	2	96.358	13.945	<0.001*	0.192
	Natural light intensity (E)	2	36.633	5.380	0.006*	0.084
	Indoor noise (F)	2	144.700	22.018	<0.001*	0.270
NA	Ceiling height (A)	2	55.258	9.427	<0.001*	0.139
	Spatial openness (B)	2	85.633	20.850	<0.001*	0.263
	Natural light intensity (E)	2	54.233	17.571	<0.001*	0.230
	Indoor noise (F)	2	32.400	6.452	0.002*	0.099

* Indicates *p* < 0.05.

**Figure 11:**
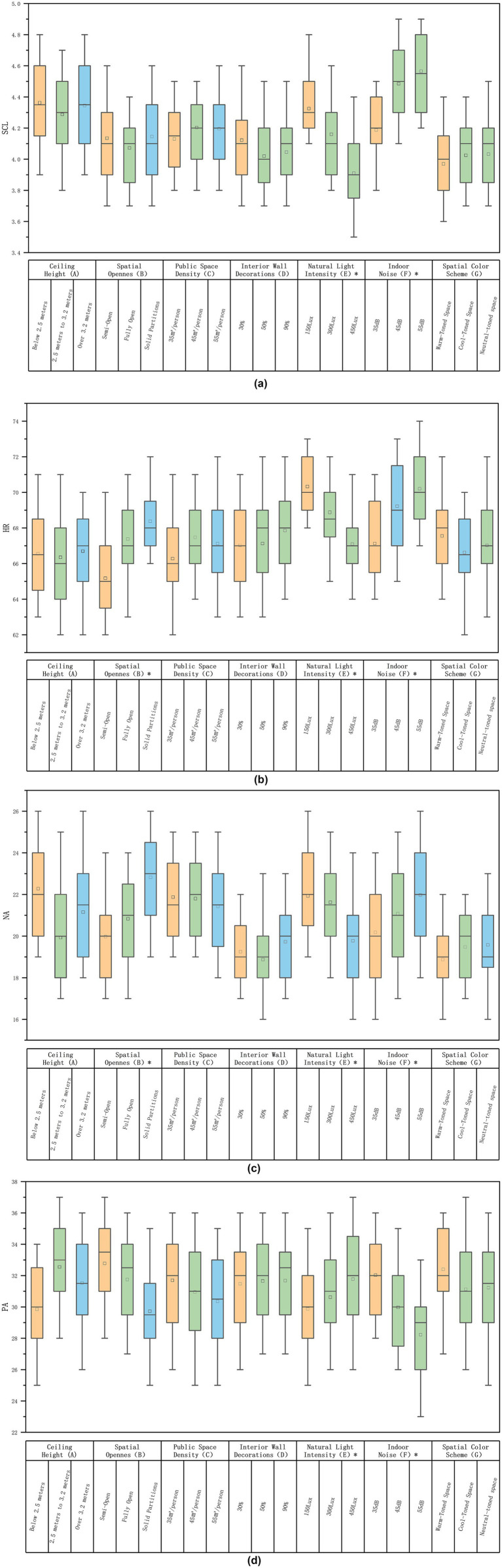
Trends in the physiological and psychological effects of different factor levels: **(a)** Trends in the effects of factor levels on SCL; **(b)** Trends in the effects of factor levels on HR; **(c)** Trends in the effects of factor levels on NA; **(d)** Trends in the effects of factor levels on PA.

Analysis of variance (ANOVA) on SCL and HR revealed that natural light exposure (E) and indoor noise (F) exerted extremely significant effects (*p* < 0.001) on both SCL and HR. Moreover, these factors yielded the largest effect sizes (η^2^ > 0.25), demonstrating their roles as the factors most strongly associated with physiological stress responses in the elderly. The trends in the effects of each factor level on skin conductance levels are shown in Figure 11a. Main effects analysis illustrated the trend of each design factor’s influence on skin conductance levels, with higher absolute values reflecting greater degrees of physiological stress. The highest physiological stress occurred under 55 dB indoor noise, while 450Lux indoor illumination proved most effective in alleviating stress. Spatial permeability (B) significantly affected only HR but not SCL, preliminarily suggesting differing sensitivities of physiological indicators to environmental stimuli. The trends in the effects of each factor level on heart rate are shown in Figure 11b. Indoor noise at 55 dB showed the strongest association with heart rate responses, while semi-open spaces were associated with the minimal effect on this physiological stress indicator.

To determine the impact of design features on psychological responses, the PANAS scale assessment was conducted following completion of the VR scene perception task to evaluate psychological reactions among elderly participants. ANOVA was performed on the PA (positive) and NA (negative) scores from the PANAS assessment. Features meeting the significance effects included: indoor ceiling height (A), spatial permeability (B), natural light intensity (E), and indoor noise levels (F). The trends in the effects of each factor level on negative ratings are shown in Figure 11c. For indoor ceiling height (A), the lowest ceiling height below 2.5 metres yielded the highest negative scores. Regarding spatial permeability (B), solid partitions generated the highest negative scores, followed by fully open spaces and semi-open spaces. With respect to natural light intensity (E) and indoor noise (F), illuminance levels of 150 lux and indoor noise levels of 55 dB were associated with the highest negative scores. The following results are presented solely as exploratory screening findings. These results are intended to generate hypotheses for future confirmatory studies rather than to provide statistically reliable causal evidence.

### 3.2 Post-hoc verification and correlation analysis

Post-hoc comparisons (Bonferroni correction) were conducted for the significant main effects identified in the aforementioned ANOVA to determine the optimal levels for each design characteristic. Bonferroni correction data are shown in Table 4.

**Table 4:** Bonferroni correction data.

Variable	Design characteristics	Numerical value	*p*-value	Post-hoc comparison
SCL	Natural light intensity (E)	4.33 ± 0.18	0.000***	E3<E1***; E3<E2***; E2<E1*
		4.16 ± 0.23		
		3.01 ± 0.26		
	Indoor noise (F)	4.19 ± 0.23	0.000***	F1<F2***; F2<F3; F1<F3***
		4.49 ± 0.22		
		4.57 ± 0.27		
HR	Spatial openness (B)	65.18 ± 2.19	0.000***	B1<B2***; B2<B3; B1<B3***
		67.38 ± 2.03		
		66.97 ± 2.40		
	Natural light intensity (E)	70.33 ± 1.91	0.000***	E3<E1***; E3<E2***; E2<E1***
		68.88 ± 2.10		
		67.10 ± 1.68		
	Indoor noise (F)	67.13 ± 2.09	0.000***	F1<F2***; F2<F3; F1<F3***
		69.22 ± 2.55		
		70.20 ± 2.00		
PA	Ceiling height (A)	29.85 ± 3.00	0.000***	A1<A2***; A3<A2; A1<A3**
		32.55 ± 2.28		
		31.53 ± 2.97		
	Spatial openness (B)	32.78 ± 2.51	0.000***	B2<B1; B3<B2***; B3<B1***
		31.75 ± 2.63		
		29.73 ± 2.75		
	Natural light intensity (E)	29.88 ± 2.54	0.006***	E1<E2; E2<E3; E1<E3***
		30.63 ± 2.35		
		31.78 ± 2.90		
	Indoor noise (F)	32.03 ± 2.62	0.000***	F3<F2; F2<F1***; F3<F1***
		29.98 ± 2.63		
		28.23 ± 2.44		
NA	Ceiling height (A)	22.28 ± 2.24	0.000***	A2<A1***; A3<A1; A2<A3*
		19.93 ± 2.35		
		21.15 ± 2.66		
	Spatial openness (B)	19.98 ± 1.86	0.000***	B1<B2; B3<B2***; B1<B3***
		20.83 ± 2.18		
		22.83 ± 2.02		
	Natural light intensity (E)	21.93 ± 1.70	0.000***	E3<E2***; E2<E1***; E3<E1***
		21.63 ± 1.66		
		19.77 ± 1.90		
	Indoor noise (F)	20.18 ± 2.42	0.002***	F1<F2; F2<F3; F1<F3***
		21.08 ± 2.33		
		21.98 ± 2.00		

*** denotes *p* < 0.01; ** denotes *p* < 0.05; * denotes *p* < 0.1.

Specifically, within the indoor ceiling height (A) dimension that significantly influenced PANAS scale assessments, the 2.5–3.2 metre ceiling height space (A2) yielded the highest PA scores and lowest NA scores. Regarding spatial openness (B) design features, solid partitions (B3) produced the highest heart rate values, while semi-open spaces (B1) elicited the highest PA scores and lowest NA scores. With respect to natural light intensity (E), SCL and HR values under 450Lux (E3) were significantly lower than those at 300Lux (E2) and 150Lux (E1). In the PANAS assessment, the 450Lux (E3) yielded significantly superior scores compared to 300Lux (E2) and 150Lux (E1). For indoor noise (F), physiological and psychological indicators, along with PANAS scores, were significantly superior at 35 dB (F1) compared to higher noise levels.

To examine the consistency between subjective psychological responses and objective physiological indicators, endeavours were also made to calculate Pearson correlation coefficients between physiological indicators (SCL, HR) and psychological indicators (PA, NA) across three design conditions. Table 5 presents the correlation matrix of physiological and psychological indicators under distinct design features. Results indicated a weak yet significant correlation between HR and positive/negative effects. For instance, under natural light conditions, HR and PA exhibited (r = −0.241, *p* < 0.01). This indicated that physiological arousal aligned with the direction of change in emotional states. However, the correlation pattern between skin conductance and psychological scale scores was relatively weaker (e.g., under spatial transparency, r = −0.006, *p* > 0.01 for SCL and PA). This demonstrated that SCL exhibited a weaker association with noise-triggered emotional alterations compared to HR in this research, suggesting that SCL and HR might capture distinct physiological dimensions of emotional experience.

**Table 5:** Correlation matrix of physiological and psychological indicators under significant design features.

Design characteristics	Variable		PA	NA
Spatial openness (B)	SCL	Pearson correlation coefficient	−0.006	0.005
		Significance level	0.815	0.958
	HR	Pearson correlation coefficient	−0.192*	0.196*
		Significance level	0.036	0.032
Natural light intensity (E)	SCL	Pearson correlation coefficient	−0.182*	0.200*
		Significance level	0.046	0.028
	HR	Pearson correlation coefficient	−0.241**	0.299**
		Significance level	0.008	0.001
Indoor noise (F)	SCL	Pearson correlation coefficient	−0.232*	0.247*
		Significance level	0.011	0.007
	HR	Pearson correlation coefficient	−0.184*	0.203**
		Significance level	0.045	0.026

*** denotes *p* < 0.01; ** denotes *p* < 0.05; * denotes *p* < 0.1.

### 3.3 Multivariable linear regression models and key predictor variables

Based on the significant factors identified by ANOVA, a predictive model was further established to determine the relative magnitude of factors with larger standardized beta coefficients in cases involving the simultaneous presence of all factors. Despite the potential for multicollinearity, which was minimized through the use of an orthogonal design, this approach was employed to construct a ranking of contribution levels. Regression results revealed indoor noise (F) as a significant variable in all four models and the strongest predictor in SCL, HR, and PA. This established noise as a core design characteristic influencing older adults’ physical and mental states, necessitating prioritised noise level control. Public space density proved significant in SCL, PA, and NA, serving as the strongest predictor in NA. Low-density design was therefore considered crucial for alleviating negative emotions and reducing psychological distress. The multivariable linear regression model is shown in Table 6.

**Table 6:** Multivariable linear regression model.

Predictor variable	SCL	HR	PA	NA
Ceiling height (A)	0.358***	−0.139**	−0.139*	0.162**
Spatial openness (B)	0.099	−0.079*	−0.126*	0.175**
Public space density (C)	0.170**	−0.081	−0.176**	0.247***
Interior wall decorations (D)	0.033	−0.030	−0.104	−0.110
Natural light intensity (E)	0.117*	0.167**	−0.205**	0.160**
Indoor noise (F)	0.455***	0.178**	−0.256***	0.155**
Spatial color scheme (G)	−0.031	−0.066	−0.106	−0.106

*** denotes *p* < 0.01; ** denotes *p* < 0.05; * denotes *p* < 0.1. Significance levels (*p*-values) are reported from exploratory ANOVA without correction for non-independence. They serve as descriptive screening indices rather than confirmatory inferential thresholds.

## 4 Discussion

### 4.1 Summary of key findings

**Figure 12:**
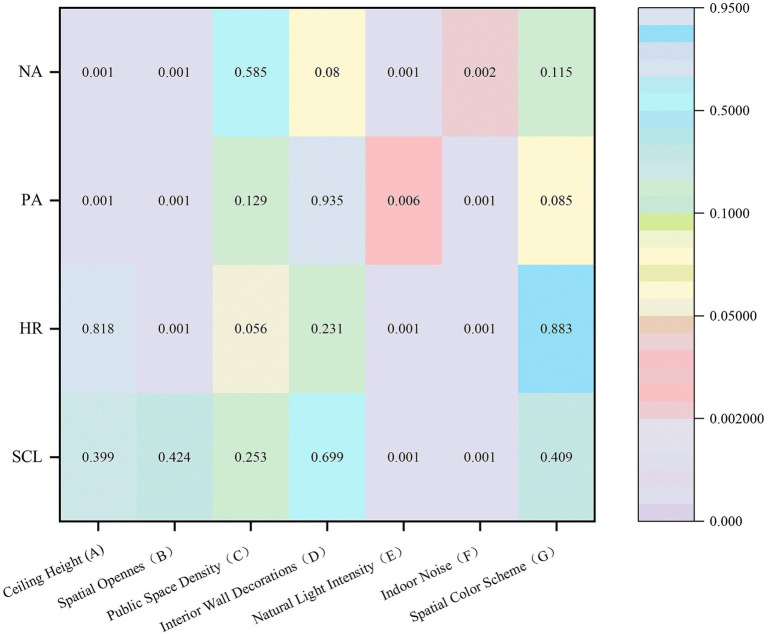
Relationship between spatial design characteristics and physiological and psychological factors.

The research findings consistently corroborate the conceptual model, indicating that spatial characteristics exert an influence on perceived stress and safety, which in turn give rise to physiological arousal and affective alterations. All the findings are confined to short-term psychophysiological responses within a virtual environment. Given that realism, presence, and ecological validity were not directly evaluated, the transferability of these results to real-world physical environments remains ambiguous. This research investigated the associations between diverse design features and the psychophysiological states of elderly individuals. Ceiling heights between 2.5 and 3.2 meters were found to most effectively alleviate psychological stress. Semi-open spaces align with the “prospect-refuge” theory, enhancing psychological security. The predictive power of public space density on negative emotions represents a preliminary finding requiring further investigation. Core design indicators include natural light intensity ≥300 Lux and indoor noise ≤35 dB, with warm and neutral color combinations proving more suitable. These findings reflect short-term associations observed in a VR environment; they do not represent verified causal mechanisms or long-term mental health impacts. Additionally, the VR experiments in this study face limitations in translating virtual experiences to reality, indicating areas for optimization in future research. The relationship between spatial design characteristics and physiological and psychological factors is shown in Figure 12.

### 4.2 Results discussion and theoretical dialogue

In accordance with environmental stress theory and perceived control theory, ceiling height (A) had a significant influence on the psychological stress levels of the elderly. The lowest stress levels were found among those in environments with ceiling heights ranging from 2.5 to 3.2 metres. Previous research has shown that higher and more open ceiling heights had a positive effect on their well – being (Iwayama et al., 2025; Presti et al., 2022). Enclosed spaces with lower ceilings often induced a sense of oppression (Vartanian et al., 2015). This suggested a correlation between individual emotional stress and indoor ceiling height, where emotional stress increased as the ceiling height decreased. Adequate ceiling height could alleviate the sense of claustrophobia among the elderly and enhance the spatial comfort. Therefore, future age-friendly design and spatial planning could primarily adopt ceiling heights of 2.5 to 3.2 metres. When renovating old residential communities, increasing the net floor height by removing non-load-bearing suspended ceilings and optimizing the layout of utility pipes could achieve the dual goals of improving spatial comfort and maximizing utilization efficiency.

Regarding Spatial Openness (B), semi-open spaces exhibited more stable HR readings than solid partitions and fully open spaces, along with higher PA scores. This aligned with the conclusion drawn from interviews that “spacious or overly enclosed spaces could easily trigger feelings of panic and oppression.” The partial visual barrier likely fostered a perceived sense of refuge and territorial control (environmental perception), which reduced the perceived uncertainty and exposure associated with fully open layouts, thereby lowering anxiety and physiological arousal. Hypothesis H2 was thus verified. In 1975, British geographer Jay Appleton put forward the “prospect-refuge” theory. This theory posits that humans’ preference for specific landscapes stems from the instincts evolved by their ancient ancestors during the struggle for survival, as individuals tend to favor sites furnishing both “refuge” and “prospect.” In this study, semi-open spaces were observed to achieve a balance between partial screening and visual transparency, creating a sense of private sanctuary while accommodating controlled visibility needs. This approach reduced anxiety stemming from spatial uncertainty. Such a spatial structure held considerable significance for age-friendly design, enhancing psychological security and environmental adaptability among the elderly, thereby further supporting the validity of Hypothesis H2. Therefore, it is recommended to adopt a semi-open layout in public activity areas, achieving a semi-open design by replacing solid partitions with greenery or wooden latticework. These elements should match the interior ceiling height to achieve approximately 50% visual permeability. This approach could ensure visual permeability and spatial connectivity while creating relatively independent, adjustable private spaces.

Public space density (C) and interior wall decorations (D) showed no statistically significant correlation in physiological and psychological measurements. However, the multivariable linear regression model indicated that public space density (C) could predict NA. A possible explanation could be that the marginal effect of public space density (C) only emerged in the regression model after controlling for other variables, yet its standalone main effect (ANOVA) failed to reach statistical significance. Consequently, this was considered a preliminary finding warranting further investigation. Previous studies confirmed the significance of low-density design for alleviating negative emotions and reducing psychological distress (Van Staden, 1984). Crowded spaces could lead to emotional stress and physical irritation (Van Staden, 1984). The WHO’s Guidelines for Healthy Housing also document that overcrowded living spaces are one of the risk factors contributing to poor mental health. In the design of age-friendly spaces, this study recommends providing ample space for older adults. Flexible arrangements of movable seating and functional zoning mitigated crowding caused by congregations; simultaneously, controlling maximum occupancy limits prevented psychological stress arising from excessive density. Wall coverings in elderly spaces could be considered occupy 30–50% of the surface area, avoiding 90% coverage which created visual clutter and a sense of oppression. Decorative elements should thus prioritise natural landscapes and soft patterns, predominantly in light colour palettes. For materials, the selection of matte, eco-friendly finishes should be recommended to minimise glare and alleviate eye strain.

Natural light intensity (E) exerted a pronounced impact on both the physiological and psychological statistics. A high-intensity lighting design of 450 lux enabled the elderly individuals to perceive a sense of ample spatial vitality, whereas a low-intensity design of 150 lux was observed to convey a dim and oppressive atmosphere, directly inducing negative emotions. This indicates a significant association between natural light intensity and physiological and psychological indicators. This finding partially aligned with the research by Zhen Xu et al., indicating that adequate indoor lighting could alleviate depression (Xu et al., 2026) while elevating serotonin and endorphin levels to improve individuals’ sleep quality (Valizadeh et al., 2015). When designing core spaces, natural light intensity should not fall below 300 lux, with 450 lux being optimal. New buildings could increase the area of south-facing windows and employ low-reflectance glass to enhance illumination levels.

Indoor noise (F) significantly affected both the physiological and psychological statistics. Excessively high noise levels were considered a health risk (Wicki et al., 2025). Cross-indicator predictions of common strong predictors revealed significant indoor noise across all four models. It emerged as the strongest predictor in SCL, HR, and PA, establishing itself as a core design feature affecting the physical and mental well-being of the elderly. Collectively, the findings of this study provide strong support for the environmental stress theory. When environmental demands, such as high noise levels and low ceiling heights, exceeded older adults’ adaptive capacities, they significantly depleted psychological resources, triggering physiological stress responses and negative emotions. Moreover, the auditory system of the elderly deteriorated, lowering their perception threshold for mid-to-low frequency noise. At an identical decibel level, the same noise produced enhanced neural stimulation. In environments below 35 dB, the sympathetic nervous system remained stable. Noise exceeding 45 dB was associated with elevated stress-related responses, and long-term exposure worsened anxiety, sleep disorders, and other problems, consistent with the findings of the WHO (Guski et al., 2017). In residential settings, noise levels within elderly living spaces should be strictly controlled below 35 dB. Building exteriors should employ integrated soundproofing and thermal insulation materials, while double-glazed insulated acoustic glass should be recommended for windows. Additionally, public areas should incorporate acoustic barriers and sound-absorbing suspended ceilings to minimise the transmission of activity-related noise.

In this study, while colour combinations (G) did not demonstrate statistically significant effects, numerical trends indicated that warm hues were more likely to evoke feelings of warmth and comfort. This aligned with previous research (Maier and Elliot, 2014; Nijdam, 2005) and warranted further ecologically grounded exploration in real-world settings. The role of neutral colours should also be further investigated, as they could exert a calming and stabilising influence on individuals’ thoughts and emotions (Al-Ayash et al., 2016). Therefore, it was hereby recommended that the colour scheme for the space incorporate warm tones such as light wood and beige, complemented by neutral shades like grey and white. Additionally, extensive use of cool colours or highly saturated hues should be avoided to prevent a sense of oppression arising from cold tones.

When examining the influence of demographic factors on outcomes, the emphasis was placed on three crucial elements: gender, age, and cognitive level. It was found that gender and age had no substantial impact on the results, and the differences in relevant physiological and psychological parameters were not statistically significant. Cognitive levels were classified into normal and mild cognitive impairment groups, and statistically significant effects were observed on both physiological and psychological statistics. The physiological values of elderly individuals in the mild cognitive impairment group were notably higher than those in the normal group. Moreover, their positive psychological scores were significantly lower compared to the normal group, while their negative psychological scores exhibited a significant increase compared to the normal group.

A crucial priority persisted in enhancing the comprehension of the constraints associated with translating virtual experiences into real-world contexts. In this regard, short-term VR exposure was employed for the construction of experimental scenarios, yet it failed to replicate the long-term effects in real-world environments. The psychological adaptation of older adults to spatial environments was still regarded as an ongoing, dynamic process. Nevertheless, short-term VR experiences faced challenges in capturing the long-term cumulative impact of spatial layouts on mental well-being. Furthermore, VR scenarios predominantly relied on visual and auditory stimuli, lacking multisensory integration, including tactile sensations, olfactory cues, and temperature. Conversely, the interaction among multiple senses in real-world spaces was found to play a pivotal role in regulating the psychological perceptions of older adults. Additionally, VR environments did not account for behavioral adaptation and social interaction within real-world spaces. Consequently, future research should concentrate on longitudinal tracking within authentic residential settings, the integration of multisensory simulation technologies, and the incorporation of behavioral and social variables, aiming to enhance the ecological validity and practical applicability of research findings.

This study has one potential limitation: the samples for the participatory design and the VR experiment were drawn from community-dwelling older adults and nursing home residents, respectively, and there are differences between the two groups in terms of daily living habits, level of autonomy, and dependence on the physical environment. The findings of this study should not be overgeneralized, nor should they be taken to fully reflect the actual spatial needs of elderly residents in care facilities. Therefore, the findings of this study should be interpreted with caution and should not be directly equated with the actual needs of elderly residents in care facilities. However, the two-stage sampling method adopted in this study represents a deliberate trade-off between the authenticity of needs and the rigor of the experiment: community-dwelling older adults are better at expressing genuine residential pain points, while nursing home residents are more suitable for standardized VR testing. The core variables examined in this study are universal spatial environmental factors that have been proven applicable to both groups; therefore, transferring the design preferences of community-dwelling older adults to institutionalized older adults is both scientifically sound and reasonable. This sampling approach has been widely adopted in the fields of environmental psychology and VR participatory design, ensuring both the generalizability of the findings and internal reliability. This is a plausible interpretation based on the observed associations. This study did not conduct formal mechanism testing, and the participatory process did not involve the co-creation of research concepts and paradigms; rather, it focused on refining user preferences, which constitutes a clear methodological limitation of this study. A key limitation related to ecological validity should be emphasized: this study did not directly measure environmental realism, sense of presence, or perceived authenticity during VR exposure. Without these assessments, it is difficult to judge how closely the virtual experience matched perceptions of real physical spaces, and whether the observed psychophysiological responses would translate to actual age-friendly environments. Therefore, the results only support short-term responses within the VR setting, rather than strong conclusions about real-world long-term design effects.

### 4.3 Practical significance

Based on the observed associations in this VR-based experimental study, the following preliminary design insights are proposed for age-friendly spaces. These insights are derived from simulated experimental evidence and should be treated as suggestive, not definitive prescriptions. Core areas (such as living rooms and activity rooms) could be considered prioritize a ceiling height of 2.5–3.2 meters, maintain indoor noise levels ≤ 35 dB, and ensure natural illumination ≥ 300 Lux. These parameters could be considered achieved through soundproofing materials, skylights, or light-guiding devices. Public activity areas feature semi-open spaces created using movable partitions and low cabinets. Designed with a density of 45–55 square meters per person to prevent overcrowding, the color scheme primarily uses warm tones (light wood and beige), complemented by neutral hues. For seniors with mild cognitive impairment, enhance the combination of “semi-open layout + low noise.” Increase environmental visibility with 300–450 lux lighting to reduce spatial adaptation anxiety. These preliminary findings are intended to support the development of conceptual designs and hypotheses for age-friendly environments. It is strongly recommended that further validation be conducted through longitudinal assessments in physical settings prior to the implementation of actual construction or renovation projects. These are preliminary design insights based on short-term VR responses. They are not evidence-based prescriptions for real-world long-term design. Since ecological validity, realism, and presence were not directly assessed, applications to physical environments require further validation.

A central limitation of this study is the statistical treatment of non-independent repeated-measures data using standard ANOVA, without adjusting for within-subject correlations. Consequently, the reported *p*-values and significance levels likely underestimate the true variability and should not be interpreted as confirmatory. The results are best understood as exploratory screening findings that can inform the selection of variables and direction of effects for future confirmatory research employing mixed-effects modeling or repeated-measures ANOVA with proper error structures.

## 5 Conclusion

### 5.1 Research summary

This study was organized and driven by a unified conceptual model from beginning to end. The entire design, measurement, and analysis were theoretically deduced to test this model. This study was carried out to examine associations between spatial layout variations in age-friendly environments on theShort-term psychophysiological responses and immediate emotional states of older adults. It aimed to address certain shortcomings of conventional research lacking a comprehensive analysis of multi-factor interactions and suffering from insufficient data objectivity. By integrating participatory design with VR technology, seven design features were identified within this exploratory participatory process through interviews with 50 older adults. Subsequently, 18 VR scenarios were constructed to measure physiological indicators and psychological scales among 40 older adults. This exploratory VR-based study provides preliminary indications that natural light intensity, indoor noise levels, and spatial openness may be associated with short-term psychophysiological responses in older adults. These findings are not confirmatory but serve as a basis for hypothesis generation. Semi-open spaces showed a potential association with higher self-reported satisfaction in this exploratory analysis. Tentative key parameters identified from this exploratory study include ceiling heights ranging from 2.5 to 3.2 meters, noise levels of ≤ 35 dB, and illumination of ≥ 300 Lux, though these require confirmatory testing in future research. This also illustrated the feasibility of integrating VR with participatory design methodologies in this field, pending further validation. In this study, SCL and HR were employed to measure the immediate arousal state of the autonomic nervous system, whilst the PANAS scale was adopted to assess the current positive/negative emotional state among elderly participants. These indicators served as critical characteristics potentially associated with mental well-being, yet they were not synonymous with mental health itself. Mental health represents a long-term, multidimensional concept encompassing emotional, cognitive, and social adaptation aspects, whereas the indicators collected in this study could only reflect short-term physiological and emotional responses to spatial environmental stimuli.

This study captures only the immediate reactions following brief exposure to VR and does not measure long-term mental health; therefore, its conclusions cannot be directly equated with the long-term mental health effects of actual residence. The study provides empirical evidence to inform the design of age-friendly spaces that enhance perceived comfort. Overall, this study offers preliminary, data-informed design parameters for age-friendly spatial design and illustrates a potential methodological direction in the in-depth integration of users into the environmental evaluation process, offering a replicable methodological blueprint for future humanistic environmental design research. The theoretical and scholarly contributions of this study are threefold: It establishes a unified theory-driven conceptual framework integrating environmental stress theory, prospect-refuge theory, sensory aging theory, and participatory design, addressing the lack of theoretical structure in prior age-friendly environmental research. It provides a multimodal, quantifiable, and replicable method to verify environmental psychology theories using VR, transforming qualitative design opinions into testable theoretical propositions. It outlines a hypothesized psychological pathway through which spatial features may influence older adults: environmental perception, physiological arousal, affective response – providing a testable framework for future research. This study proposed a structured transformation pathway from participatory design to empathy mapping to the double-loop model to variable quantification. Through transparent coding logic, consensus rules, and reliability testing, it has demonstrated one approach to transforming users’ qualitative needs into experimental variables, with the aim of enhancing transparency and replicability, thereby strengthening the ecological validity and user-centeredness of VR experiments. This study provides exploratory evidence suggesting associations between spatial factors and short-term psychophysiological responses in a VR setting; it does not formally test causal mechanisms or long-term explanatory pathways. The observed relationships support plausible interpretations but do not constitute empirically verified mechanisms.

### 5.2 Advantages and limitations

This study has two notable strengths: First, the research methodology is rigorous. Through interviews with elderly participants and the development of an empathy framework, the study identified design features that influence the physiological and psychological states of older adults, laying a solid foundation for subsequent VR experiments. Second, the analysis is in-depth: post-hoc tests were conducted on significant design features identified through analysis of variance (ANOVA), validating the consistency of multimodal data. However, this study has several limitations: it examined only seven predefined spatial design features and did not include important factors such as window views, natural elements, and material textures, limiting the model’s comprehensiveness. The entire study was conducted in a VR environment with short-term exposure, providing only audiovisual stimuli. It lacked multisensory inputs such as tactile, olfactory, and thermal sensations, making it impossible to fully replicate real physical environments and long-term residential experiences, resulting in insufficient ecological validity. Furthermore, the VR experiment included only 40 elderly participants, a limited sample size that struggles to represent the diverse socioeconomic, residential, and health backgrounds of the elderly population, thereby limiting the generalizability of the conclusions. This study has a significant limitation affecting its internal consistency: the qualitative needs assessment sample consisted of community-dwelling older adults, while the VR experiment sample comprised institutionalized older adults. These two groups exhibit conceptual differences in terms of life autonomy, environmental dependence, spatial usage habits, and spatial expectations. Although there are certain commonalities in basic sensory preferences—such as spatial lighting, acoustic environment, height, and openness—the transferability of preferences between the two groups has not been empirically tested; This limitation may introduce a bias between the selected experimental variables and the actual needs of institutionalized older adults, thereby affecting the study’s internal consistency and ecological validity. The study only observed cross-sectional correlations and did not validate long-term causal mechanisms or formal explanatory pathways; the results reflect only short-term psychophysiological responses and cannot be equated with long-term mental health outcomes. Furthermore, due to limitations in the physiological tolerance of the elderly participants and the duration of the experiment, formal modeling analysis of multifactorial interactions was not conducted, resulting in an insufficient elucidation of synergistic influence mechanisms.

### 5.3 Future research prospects

This study has demonstrated the feasibility of VR technology in research regarding age-friendly environments. Future work can overcome technical limitations to enhance the validity of experiments. To address the current limitation of VR environments relying solely on visual stimuli, multi-sensory interaction technologies can be incorporated. For example, odour generators can be integrated, and haptic feedback devices can be configured. This creates a multi-dimensional immersive experience that encompasses sight, smell, and touch, accurately reproducing the perceptual details of real-world spaces. Simultaneously, developing age-friendly VR devices—featuring lightweight headsets, adjustable field-of-view settings, and optimized anti-motion sickness algorithms—can mitigate the impact of VR-induced motion sickness (VIMS) on older adults and broaden sample applicability. Research design could be considered transcend the limitations of cross-sectional studies by conducting long-term longitudinal tracking experiments. In the future, based on the core main effect factors identified in this study (natural light intensity, indoor noise, spatial openness, and ceiling height), the range of variables can be narrowed down. By employing a full-factorial or factorial design, formal modeling can be conducted to explore potential causal links and interaction associations among these core factors, thereby refining the analysis of the synergistic mechanisms among multiple factors in age-friendly spaces. Select communities that have completed age-friendly renovations and use VR technology to record physiological and psychological data at different stages—before, during, and after the renovation. Analyze the sustained effects of spatial layout changes on mental health, distinguishing between short-term and long-term impacts to provide evidence for evaluating the long-term efficacy of age-friendly design. Additionally, real-time physiological metrics from daily life can be collected via wearable devices, cross-validating VR experimental data to enhance ecological validity. In addition, the same group of older adults will be used to complete the entire process of participatory design and VR experiments, thereby eliminating the impact of sample differences and further enhancing the internal validity of the study.

Enhanced interdisciplinary collaboration is required for research into age-friendly environments. This involves combining theories and technologies from subjects such as environmental psychology, geriatric medicine, computer science and architecture. For instance, collaborate with geriatric medicine to explore the auxiliary intervention role of spatial layout in managing chronic conditions among the elderly. By analyzing the synergistic regulatory mechanisms of designs such as meditation spaces and rehabilitation gardens on physiological indicators and psychological states, provide support for the design of integrated medical and care communities. Collaboration with artificial intelligence can develop predictive models for elderly mental health using VR experiments and daily behavioral data. By employing machine learning to identify patterns that link spatial design to mental well-being, real-time psychological state alerts and intelligent recommendations for spatial interventions can be achieved. Furthermore, a digital twin system for age-friendly spaces can be developed to simulate spatial efficacy across diverse scenarios (such as public space crowding levels and emergency evacuation efficiency), thereby supporting dynamic spatial optimization. Future studies should incorporate standardized measures of environmental realism, sense of presence, and ecological validity to clarify the transferability of VR-based findings to physical age-friendly environments. Ultimately, this will propel age-friendly design from an “experience-driven”to a “data-driven”approach, contributing to the development of a healthy ageing society.

## Data Availability

The original contributions presented in the study are included in the article/Supplementary material, further inquiries can be directed to the corresponding author.
