# Supplementary material for: A structured participatory preference-tuning and VR-based framework for age-friendly spatial configuration: quantifying short-term psychophysiological responses and immediate affective states through dynamic virtual environment adaptation
Source: Front Psychol. 2026 Jun 11;17:1826218. doi: 10.3389/fpsyg.2026.1826218 (PMC13293921; doi:10.3389/fpsyg.2026.1826218)
Supplement: Supplementary file 1 [file data_sheet_1.docx]

Appendix A

The system records the number of operations performed by elderly users, the time spent lingering and the voice keywords they use during VR device operation.

**Table A1.** The number of operations performed.

| Physical environmental characteristics | number of operations performed | proportion |
| --- | --- | --- |
| Lighting, Noise | 3 | 25% |
| Spatial Layout | 5 | 41.6% |
| Spatial scale, Social density | 2 | 16.7% |
| Natural decor, Colour coordination | 2 | 16.7% |

**Table A2.** The time spent lingering.

| Physical environmental characteristics | Time spent lingering | proportion |
| --- | --- | --- |
| Lighting, Noise | 8s | 16% |
| Spatial Layout | 17s | 34% |
| Spatial scale, Social density | 15s | 30% |
| Natural decor, Colour coordination | 10s | 20% |

**Table A3.** The voice keywords.

| Physical environmental characteristics | The voice keywords |
| --- | --- |
| Lighting, Noise | Draw closer to the sunlight, open the curtains, close the windows, reduce the noise level. |
| Spatial Layout | Place the sofa closer to the window; ensure furniture does not obstruct movement. |
| Spatial scale, Social density | The furniture should not be too sparse, and there should be less foot traffic. |
| Natural decor, Colour coordination | Add some decorative artwork to the walls; avoid bare white walls; incorporate warm-toned furniture; remove the tubular steel chairs. |

Appendix B

| **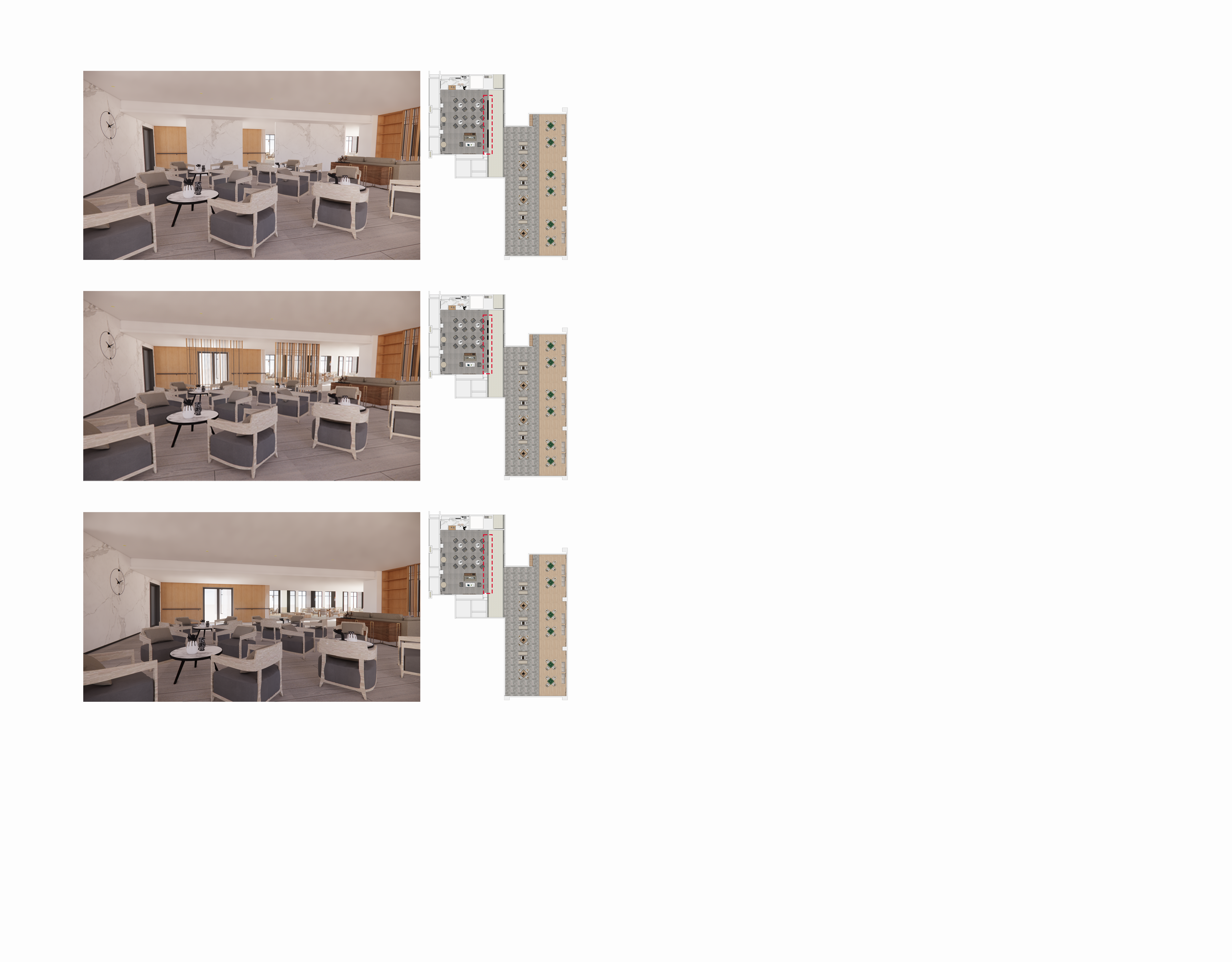** | Panel B: Semi-Open (B1)  Visual Permeability: Approx 50% openness  Partition Type: Wooden Grille  Partition Height: 3 m  Key dimension illustration: Partial Visual Connection |
| --- | --- |
| **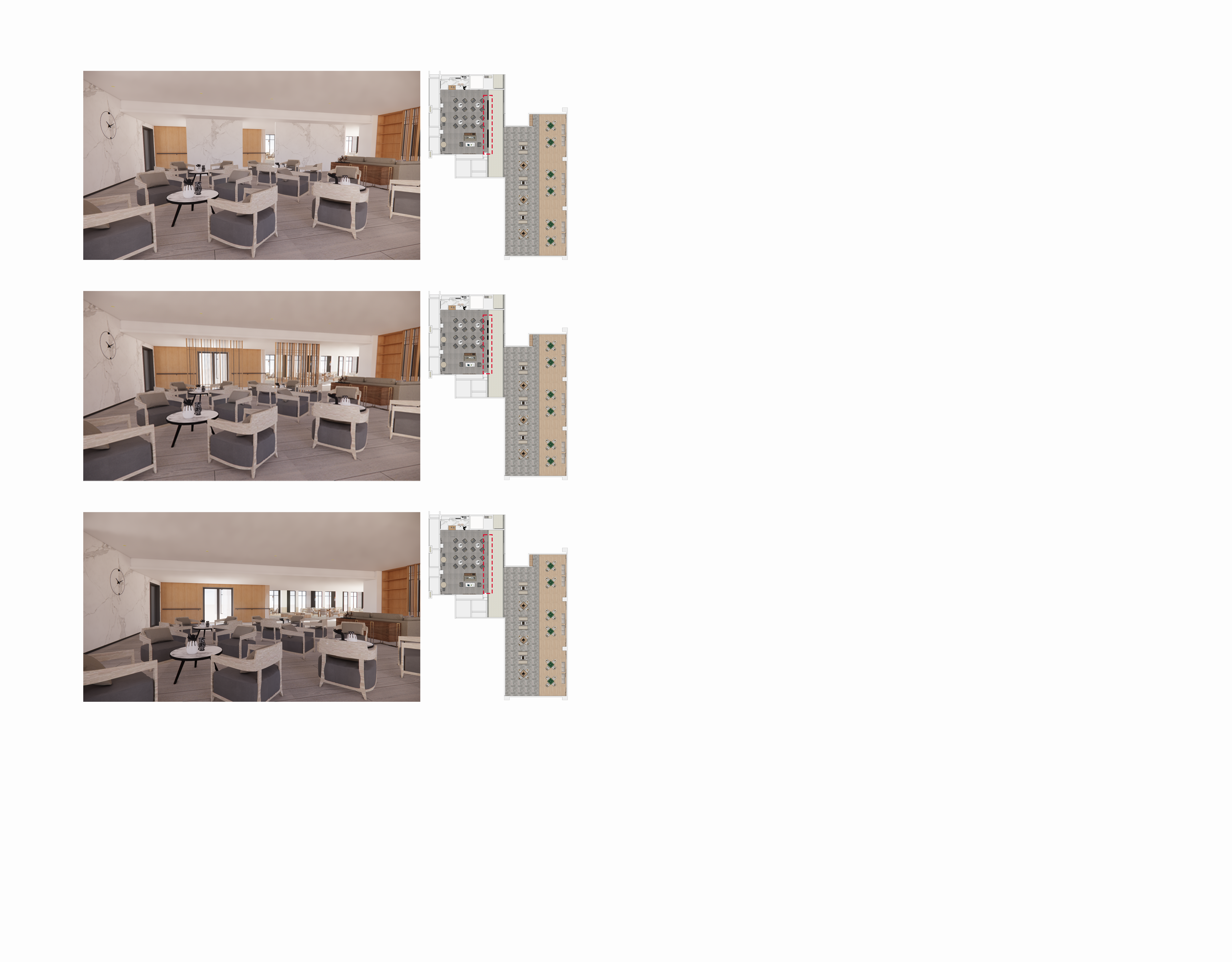** | Panel A: Fully Open (B2)  Visual Obstruction: None  Partition Height: 0 m  Transparency Ratio: 100%  Key dimension illustration: Clear Sightline |
| **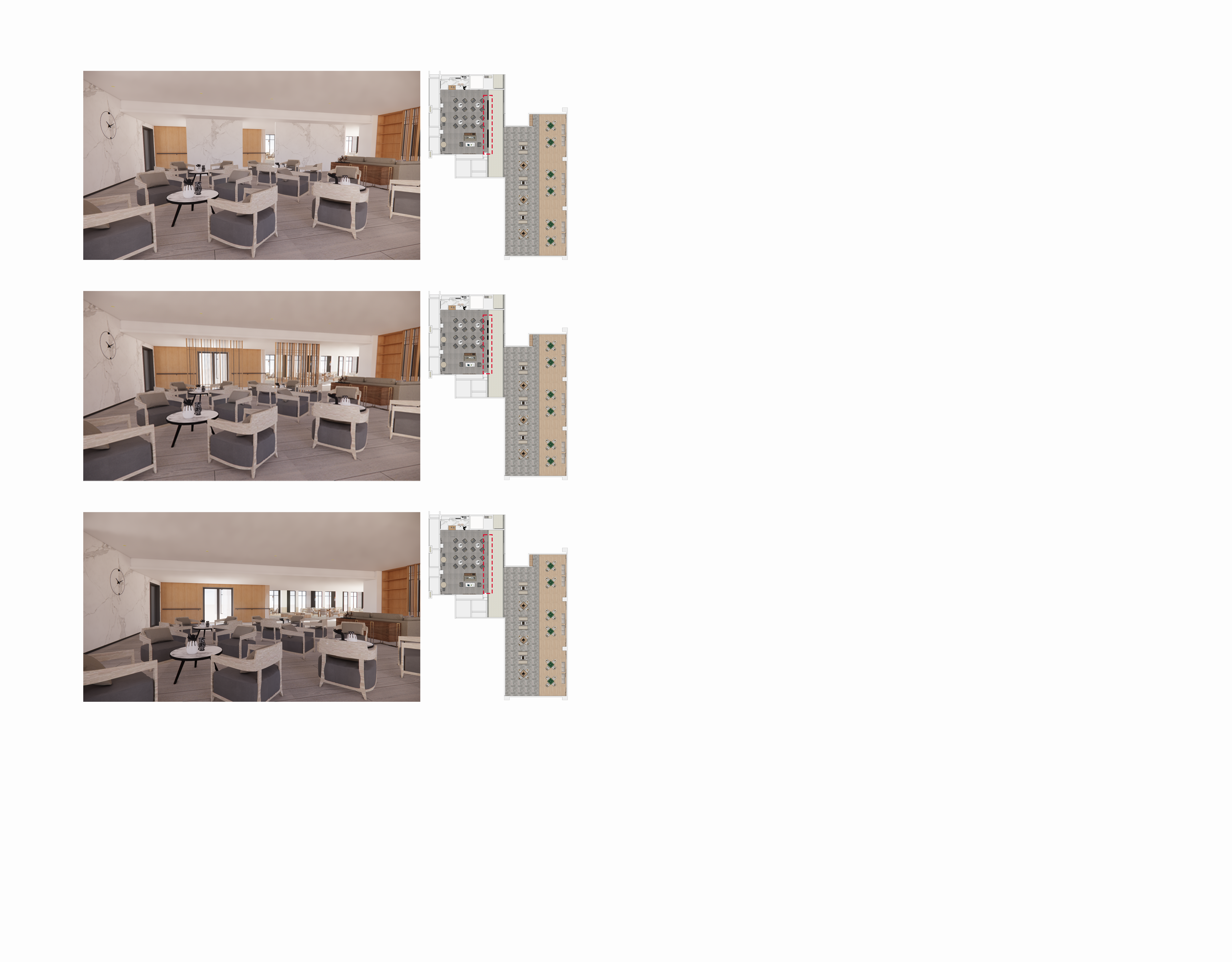** | Panel C: Solid Partitions (B3)  Visual Permeability: 0%  Partition Type: Solid Wall  Partition Height: Full Ceiling Height(3 m)  Key dimension illustration: No Visual Connection |

Figure: Operationalization of the three levels of Spatial Openness (B). (A) Semi-Open (B1): defined by partial-height partitions (approximately 50% visual permeability) that allow limited visual connection.(B) Fully Open (B2): defined as the absence of any visual barriers. (C) Solid Partitions (B3): defined by floor-to-ceiling opaque walls that completely block visual access. Schematic plan view illustrating the conceptual differences among the three levels.

Appendix C

The SCL ideal data model.

**Figure A1**


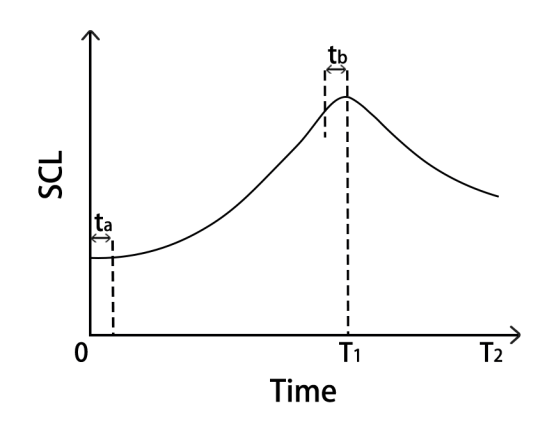


Appendix D

The original PANAS scale.

**Table A4.** Positive and Negative Affect Schedule Questionnaire.

| Indicate the extent you have felt this way in this scene: | | | | | |
| --- | --- | --- | --- | --- | --- |
| Very Slightly or not at all – 1, A little – 2, Moderately – 3, Quite a bit - 4, Extremely – 5. | | | | | |
| Items | Feeling | Scoring | Items | Feeling | Scoring |
| 1 | Happy | 1 2 3 4 5 | 10 | Irritable | 1 2 3 4 5 |
| 2 | Distressed | 1 2 3 4 5 | 11 | Nervous | 1 2 3 4 5 |
| 3 | Excited | 1 2 3 4 5 | 12 | Ashamed | 1 2 3 4 5 |
| 4 | Energetic | 1 2 3 4 5 | 13 | Elated | 1 2 3 4 5 |
| 5 | Guilty | 1 2 3 4 5 | 14 | Delightful | 1 2 3 4 5 |
| 6 | Scared | 1 2 3 4 5 | 15 | Nervous | 1 2 3 4 5 |
| 7 | Annoyed | 1 2 3 4 5 | 16 | Graceful | 1 2 3 4 5 |
| 8 | Enthusiastic | 1 2 3 4 5 | 17 | Trembling | 1 2 3 4 5 |
| 9 | Proud | 1 2 3 4 5 | 18 | Active | 1 2 3 4 5 |

Scoring Method of PANAS: Positive Affect Score (PA): Add the scores on items 1, 3, 4, 8, 9, 13, 14, 16, and 18. Scores can range from 9 - 45, with higher scores representing higher levels of positive affect; Negative Affect Score (NA): Add the scores on items 2, 5, 6, 7, 10, 11, 12, 15, and 17. Scores can range from 9 - 45, with lower scores representing lower levels of negative affect.

Appendix E

Design characteristics with significant effect.

**Table A5.**

| Variable | Design Characteristics | Df | Mean Square | F | P-value | η² |
| --- | --- | --- | --- | --- | --- | --- |
| SCL | Ceiling Height (A) | 2 | 0.062 | 0.927 | 0.399 | 0.016 |
|  | Spatial Opennes（B） | 2 | 0.062 | 0.865 | 0.424 | 0.015 |
|  | Public Space Density（C） | 2 | 0.064 | 1.390 | 0.253 | 0.022 |
|  | Interior Wall Decorations（D） | 2 | 0.488 | 0.710 | 0.699 | 0.055 |
|  | Natural Light Intensity（E） | 2 | 1.746 | 33.901 | ＜0.001* | 0.367 |
|  | Indoor Noise（F） | 2 | 1.583 | 27.781 | ＜0.001* | 0.322 |
|  | Spatial Color Scheme（G） | 2 | 0.047 | 0.901 | 0.409 | 0.151 |
|  |  |  |  |  |  |  |
| HR | Ceiling Height (A) | 2 | 1.233 | 0.201 | 0.818 | 0.003 |
|  | Spatial Opennes（B） | 2 | 107.200 | 26.321 | ＜0.001* | 0.310 |
|  | Public Space Density（C） | 2 | 15.233 | 2.959 | 0.056 | 0.048 |
|  | Interior Wall Decorations（D） | 2 | 8.108 | 1.486 | 0.231 | 0.025 |
|  | Natural Light Intensity（E） | 2 | 104.358 | 28.746 | ＜0.001* | 0.330 |
|  | Indoor Noise（F） | 2 | 98.775 | 19.934 | ＜0.001* | 0.254 |
|  | Spatial Color Scheme（G） | 2 | 0.355 | 0.506 | 0.883 | 0.044 |
|  |  |  |  |  |  |  |
| PA | Ceiling Height (A) | 2 | 74.308 | 9.671 | ＜0.001* | 0.142 |
|  | Spatial Opennes（B） | 2 | 96.358 | 13.945 | ＜0.001* | 0.192 |
|  | Public Space Density（C） | 2 | 18.300 | 2.088 | 0.129 | 0.034 |
|  | Interior Wall Decorations（D） | 2 | 0.475 | 0.067 | 0.935 | 0.001 |
|  | Natural Light Intensity（E） | 2 | 36.633 | 5.380 | 0.006* | 0.084 |
|  | Indoor Noise（F） | 2 | 144.700 | 22.018 | ＜0.001* | 0.270 |
|  | Spatial Color Scheme（G） | 2 | 20.108 | 2.522 | 0.085 | 0.041 |
|  |  |  |  |  |  |  |
| NA | Ceiling Height (A) | 2 | 55.258 | 9.427 | ＜0.001* | 0.139 |
|  | Spatial Opennes（B） | 2 | 85.633 | 20.850 | ＜0.001* | 0.263 |
|  | Public Space Density（C） | 2 | 2.325 | 0.539 | 0.585 | 0.009 |
|  | Interior Wall Decorations（D） | 2 | 7.258 | 2.575 | 0.080 | 0.042 |
|  | Natural Light Intensity（E） | 2 | 54.233 | 17.571 | ＜0.001* | 0.230 |
|  | Indoor Noise（F） | 2 | 32.400 | 6.452 | 0.002* | 0.099 |
|  | Spatial Color Scheme（G） | 2 | 5.733 | 2.206 | 0.115 | 0.036 |
